# Socio-emotional well-being is associated with cognitive function and informant-rated cognitive decline: Results from the Harmonized Cognitive Assessment Protocol in Europe

**DOI:** 10.1017/S1355617726101933

**Published:** 2026-03-27

**Authors:** Martina Luchetti, Antonio Terracciano, Selin Karakose, Damaris Aschwanden, Yannick Stephan, André Hajek, Angelina R. Sutin

**Affiliations:** 1 Behavioral Sciences and Social Medicine, Florida State University College of Medicine, USA; 2 Geriatrics, Florida State University College of Medicine, USA; 3 Institute for Ageing Research, Eastern Switzerland University of Applied Sciences - Campus St Gallen, Switzerland; 4 EuroMov, University of Montpellier, France; 5 Health Economics and Health Services Research, University Medical Center Hamburg-Eppendorf, Germany

**Keywords:** Cognition, moderation, life satisfaction, loneliness, meaning in life, social connectedness

## Abstract

**Objective:**

This study examines how multiple dimensions of socio-emotional well-being relate to cognitive functioning in older adults, and whether the associations vary by cognitive status, depression, and socio-demographic factors.

**Methods:**

Data from the Harmonized Cognitive Assessment Protocol of the Survey of Health, Ageing and Retirement in Europe (*n* = 2,650; mean age = 76; 54.5% females) were used to test associations between life satisfaction, meaning in life, social connectedness, and loneliness with global, domain-specific cognitive performance, and informant-rated cognitive decline.

**Results:**

Linear mixed models, with individuals nested within five countries, found that higher life satisfaction, meaning in life, and social connectedness were associated with better cognitive outcomes, whereas greater loneliness was associated with worse performance and greater informant-rated decline. The largest effect sizes were observed for meaning in life (median *β* = .10) and loneliness (median *β* = −.09) across cognitive measures. The associations generally remained significant adjusting for well-known clinical (e.g., diabetes), behavioral (e.g., physical inactivity), and psychological (depressive symptomatology) risk factors for dementia. Moderation and sensitivity analyses suggested that associations with global cognition hinged on the inclusion of participants classified with cognitive impairment, while some domain-specific associations (e.g., loneliness and episodic memory) were observed only in individuals without cognitive impairment. Overall, evidence for moderation by cognitive status, depression and age was limited, and no moderation was observed for sex or education.

**Conclusions:**

The results underscore the importance of socio-emotional well-being in cognitive aging and highlight the need for longitudinal research to clarify mechanistic pathways and inform targeted interventions.

## Statement of Research Significance

**Research Question(s) or Topic(s):** This study examined how dimensions of socio-emotional well-being – life satisfaction, meaning in life, social connectedness, loneliness – are associated with cognitive functioning in older adults, and whether associations vary by cognitive status, depression, or socio-demographic factors. **Main Findings:** Higher meaning in life, life satisfaction, and social connectedness were associated with better global and domain-specific cognitive performance, while higher loneliness was linked to poorer cognitive outcomes and informant-rated cognitive decline. Associations with global cognition hinged on participants with mild to severe cognitive impairment; some domain-specific associations (e.g., loneliness and episodic memory) were observed only in unimpaired individuals. Most associations were not moderated by cognitive status, depression, or socio-demographics. **Study Contributions:** This study offers new evidence on the importance of socio-emotional well-being in cognitive aging. It highlights the potential value of interventions that enhance well-being to help preserve cognitive health, particularly in individuals at higher risk of dementia.

Over 55 million adults worldwide have Alzheimer’s disease or another dementia – a number that is projected to almost triple by 2,050 due to the aging of world population (World Health Organization, 2023). Even with emergent drug therapies (Alzheimer’s Association, 2025), *risk reduction* remains a key focus of the World Health Organization (WHO)’s action plan response to dementia (World Health Organization, 2017, 2024). The WHO recommends an intense effort to identify modifiable risk factors, including social and psychological factors, that can be targeted in interventions to prevent or delay onset of the disease. Among these factors, depression and social isolation have received the most attention and are widely recognized as contributors to increased risk of dementia and cognitive impairment (Livingston et al., 2024). There are, however, other aspects of social and emotional well-being that contribute to cognitive function, even before the onset of cognitive impairment (Sutin, Luchetti et al., 2024).

In this context, studies using data from the Harmonized Cognitive Assessment Protocol (HCAP) – a comprehensive, cross-national neuropsychological assessment designed to support harmonized, population-based research on aging and cognitive health (Langa et al., 2020; Zhang et al., 2024) – have identified some critical socio-emotional dimensions related to cognitive performance in older adults. For instance, feelings of dissatisfaction with one’s social relationships, commonly referred as *loneliness*, have been associated with poor performance across multiple cognitive domains (episodic memory, speed-attention, visuo-spatial abilities, numeric reasoning, verbal fluency) and cognitive decline rated by a knowledgeable informant (e.g., a spouse; Lee et al., 2025). Notably, the association between loneliness and cognition replicated across diverse HCAP samples and held controlling for depressive symptomatology and indices of social isolation, such as living alone (Lee et al., 2025). In addition to loneliness, other dimensions of *social connectedness* have also been associated with cognitive outcomes in HCAP-based studies (Katz et al., 2020; Kim & Pan, 2025; Meister & Zahodne, 2022). In general, larger social networks or frequent social contacts were linked to better cognitive performance, while perceived strain in social relationships was associated with worse cognitive scores (Meister & Zahodne, 2022). Still, mixed or null results have also been reported in other studies that examined loneliness and social connectedness (Kyröläinen & Kuperman, 2021; Samtani et al., 2022; Solé-Padullés et al., 2022; Windsor et al., 2020), with associations varying based on the sample composition, the type of social relationship/measure, and the cognitive domain assessed.

The broader literature highlights other well-being indicators as potential protective factors for cognitive health. For instance, higher *satisfaction with life* has been associated with maintaining spatial cognition, processing speed, and verbal working memory over time (Zainal & Newman, 2022). Similarly, having *meaning in life*, a sense that one’s life has coherence, purpose, and significance, has been linked to better performance in episodic memory and verbal fluency tasks (Sutin et al., 2022), which are sensitive markers for risk of future cognitive impairment (Josefsson et al., 2023; Sutin et al., 2019). While some of these associations may be considered well-established, testing their generalizability using contemporary data can evaluate their relevance across different populations and evolving contexts. Furthermore, few studies have examined how different dimensions of well-being contribute to both global cognition and domain-specific cognitive performance within a single sample (though see Pfund et al., 2025; Sutin et al., 2018; Sutin et al., 2024), limiting a comprehensive understanding of the relationship between socio-emotional well-being and cognition and the ability to pinpoint specific well-being and cognitive dimensions to target in interventions.

The present work builds on and extends prior research on socio-emotional well-being and cognitive health in several ways. First, guided by the Positive Health framework (Ryff et al., 2004; Seeman, 1989), we adopt a multidimensional approach to investigate how various aspects of socio-emotional well-being relate to cognitive function in a large, multi-national sample of older adults. Specifically, we leverage data from the HCAP sub-study of the Survey of Health, Ageing and Retirement in Europe (SHARE) to examine how life satisfaction, meaning in life, social connectedness, and loneliness are associated with global and domain-specific cognitive performance and informant-rated cognitive decline. This latter measure is particularly valuable, as informants may detect everyday cognitive difficulties that standardized testing can miss (Pérez-Blanco et al., 2022). Second, we examine whether the associations between these socio-emotional dimensions and cognitive outcomes are moderated by participants’ cognitive status (e.g., do the associations differ among individuals who have a mild to severe cognitive impairment compared to those without an impairment?) and presence/absence of depressive symptomatology (e.g., is socio-emotional well-being still relevant for cognition among individuals with depression?). Third, we assess whether the associations are independent of clinical (e.g., diabetes) and behavioral (e.g., physical inactivity) factors known to be associated with increased risk of dementia (Livingston et al., 2024), and whether they vary by age, sex, and education. Although evidence suggests that the associations between dimensions such as loneliness with cognitive functioning are largely independent of cognitive status or depression, and remain relatively consistent across age, sex, and education groups (Lee et al., 2025), it is essential to empirically evaluate the generalizability of these relationships across diverse populations. Examining potential moderators, both clinical and socio-demographic, will help identify groups most likely to benefit from well-being interventions and advance understanding of how socio-emotional resources support cognitive resilience in later life. In line with prior research, we hypothesize that individuals with higher socio-emotional well-being will perform better across multiple cognitive domains and have less informant-rated cognitive decline. However, we do not specify hypotheses for moderation by cognitive status, depression, or socio-demographic factors.

## Methods

### Participants and procedure

Participants completed the HCAP, a sub-study of the SHARE (doi: 10.6103/SHARE.HCAP1.100). The protocol was administered in five SHARE countries (Czech Republic, Denmark, Germany, France, Italy) among respondents aged 65 years and older who participated in at least one of the last three regular waves of SHARE (Waves 6, 7, or 8) and were eligible for Wave 9 (Bergmann et al., 2024; Börsch-Supan et al., 2025). The sampling strategy targeted individuals with low scores on the regular SHARE cognitive module to oversample individuals at high risk for cognitive impairment or dementia (Börsch-Supan et al., 2025). Of 3,546 eligible individuals, 2,687 participated in the HCAP study (Börsch-Supan et al., 2025). The protocol was administered between May and November 2022, approximately five months after Wave 9 data collection. In addition to the cognitive assessment, participants nominated one knowledgeable close other to report on their cognitive function. Data collection was performed in accordance with Helsinki Declaration; written informed consent was obtained from all individuals, and the protocol was approved by the Ethics Committee of the Max Planck Institute in Germany.

For the current analysis, participants were selected if they had data available on socio-emotional well-being (life satisfaction, meaning in life, social connectedness, or loneliness), information on age, sex, and education, and completed at least one measure of the HCAP assessment. We included all participants with at least one cognitive measure to maximize data use and avoid excluding respondents with cognitive difficulties or impairment who were more likely to not complete every task. The analytical sample consisted of a total 2,650 participants across the five SHARE-HCAP countries (Czech Republic, *n* = 483; Denmark, *n* = 566; France, *n* = 520; Germany, *n* = 546; Italy, *n* = 535). Most respondents (79.9%) completed all HCAP measures or only missed one. Descriptive statistics are in Table 1.

**Table 1.** Descriptive statistics

Variables	M (SD) or %	Missing	ICC
Age (years)	76.29 (7.47)	0	.017
Range	65–102		
Sex (female)	54.5 (1,443)	0	
Education (range 0–6)	2.93 (1.50)	0	.223
None	3.1 (83)		
Primary education	18.6 (494)		
Primary education or first stage of basic education	14.6 (388)		
Lower secondary or second stage of basic education	36.4 (964)		
Post-secondary non-tertiary	1.5 (41)		
First stage of tertiary education	24.5 (650)		
Second stage of tertiary education	1.1 (30)		
Life satisfaction (range 0–10)	7.93 (1.65)	47	.064
Meaning in life (range 1–4)	3.57 (.71)	64	.051
Social connectedness (range 0–4)	2.12 (.85)	151	.057
Loneliness (range 1–3)	1.37 (.50)	16	.085
Depression (yes)	24.7 (655)	177	
Cognitive status		178	
Normal cognition	63.2 (1,674)		
Mild cognitive impairment	20.6 (546)		
Severe cognitive impairment	9.5 (252)		
Health-related limitations (yes)	53.0 (1,405)	3	
Hypertension (yes)	51.7 (1,369)	6	
Diabetes (yes)	18.0 (476)	6	
Obesity (yes)	23.2 (614)	47	
Ever smoked (yes)	42.0 (1,113)	2	
Physical Inactivity (yes)	17.8 (472)	2	
*Cognitive Measures*			
MMSE (range 0–30)	27.06 (3.62)	0	.115
Episodic memory (Composite Score)^a^	−.04 (.79)	5	.132
CERAD Immediate word recall (range 0–30)	16.02 (5.91)	25	.155
CERAD Delayer word recall (range 0–10)	4.67 (2.81)	32	.150
CERAD Recognition (range 0–20)	17.50 (3.14)	46	.157
Logical memory, long story immediate recall (range 0–25)	8.32 (4.92)	36	.041
Logical memory, long story delayed recall (range 0–25)	6.20 (5.04)	53	.059
Logical memory, long story recognition (range 0–15)	10.62 (2.31)	575	.048
Logical memory, short story immediate recall (range 0–6)	3.67 (1.57)	20	.058
Logical memory, short story delayed recall (range 0–6)	2.60 (1.93)	53	.042
Speed-attention (Composite Score)^a^	−.06 (.84)	32	.171
Symbol cancellation (max 60)	33.94 (14.86)	164	.110
Backward counting (max 100)	28.17 (11.31)	70	.101
Symbol digit modality test (max 110)	30.15 (14.14)	257	.113
Trail Making A (seconds)	71.89 (50.07)	188	.170
Trail Making B (seconds)	145.70 (70.44)	518	.070
Visuo-spatial abilities (Composite Score)^a^	−.03 (.89)	40	.102
Constructional praxis, immediate recall (range 0–11)	9.35 (2.20)	61	.066
Constructional praxis, delayed recall (range 0–11)	7.01 (3.45)	96	.048
Raven Matrixes (range 0–17)	13.01 (3.61)	109	.121
Numeric reasoning (HRS number series)	524.50 (32.63)	411	.074
Verbal fluency (animal naming)	20.24 (7.81)	10	.181
*Informant Measures*			
Informant-rated cognitive decline (Composite Score)^a^	.02 (.92)	0	.037
Jorm IQ-CODE (range 1–5)	3.22 (.41)	2	.019
CIS-D (range 0–15)	2.67 (2.95)	0	.050
Blessed Dementia Rating Scale – Part 1 (range 0–8)	.75 (1.28)	346	.044
10/66 (range 0–5)	.84 (1.27)	1	.016
Informant age (range 18–98)	65.07 (13.25)	18	
Informant sex (female)	67.3 (1,481)	0	
Informant education (range 0–6)	3.24 (1.33)	8	
None	1.1 (25)		
Primary education	8.7 (191)		
Primary education or first stage of basic education	14.6 (322)		
Lower secondary or second stage of basic education	45.7 (1,007)		
Post-secondary non-tertiary	.3 (6)		
First stage of tertiary education	28.2 (622)		
Second stage of tertiary education	1.0 (21)		
Relationship with respondent (spouse)	54.6 (1,202)	0	
Living together with respondent (yes)	58.0 (1,277)	0	

*Note:* Total *N* = 2,650 (Czech Republic = 483; Denmark = 566; France = 520; Germany = 546; Italy = 535); *N* = 2,202 had informant ratings. Intraclass coefficient correlation (ICC) reflects between-country variability, and it is reported for age, education, socio-emotional well-being, cognitive and informant measures.

a For domains with multiple tasks, we calculated a composite score; scores for each task were *z*-standardized (*M* = 0, SD = 1) and the mean was taken across tasks.

See supplementary material for descriptive statistics by cognitive status.

### Measures

We examined socio-emotional well-being measures that were available as part of the SHARE HCAP or Wave 9 regular assessment. A detailed description of all well-being and cognitive measures is in supplementary material (Tables S1–S3).

#### Independent variables

*Life satisfaction*. Participants responded to the question “On a scale from 0 to 10, where 0 means completely dissatisfied and 10 means completely satisfied, how satisfied are you with your life?” This single-item measure has been widely used in psychological and cross-cultural aging studies (Bartram, 2021; Tomini et al., 2016) and perform similar to multi-item scales (Cheung & Lucas, 2014). Higher scores reflect greater life satisfaction.

*Meaning in Life.* Participants responded to the question “How often do you feel that your life has meaning?” (from 1 = *often* to 4 = *never*) from the Control-Autonomy-Pleasure-Self-realization scale of quality of life in older adulthood (Gruber et al., 2024; Hyde et al., 2003). This single-item measure has been used in previous research on meaning in life, including SHARE-based studies (Sutin et al., 2022). The response scale was reverse scored to reflect higher meaning in life.

*Social connectedness.* A validated composite score (Litwin & Stoeckel, 2016; Paiva et al., 2023) was used to measure social connectedness based on multiple questions about social relationships. The measure incorporates five main characteristics of social networks within a single indicator (Gruber et al., 2024): (1) the number of persons in the network (network size), and, among this network, (2) the number of network members living within 25 km (proximity), (3) the number of network members with weekly or more contact (contact frequency), (4) the number of network members with very or extremely close emotional ties (support), and (5) the number of different types of relationships present within the network (diversity). The raw score (range 0–20) was converted to a composite scale that ranged from 0 to 4 (0 = 0; 1 = 1–5; 2 = 6–10; 3 = 11–15; 4 = 16–20); higher scores reflect greater social connectedness (Gruber et al., 2024).

*Loneliness.* The 3-item version of the R-UCLA Loneliness scale was used to measure loneliness (Gruber et al., 2024; Hughes et al., 2004). Respondents responded to the questions “How often do you feel … isolated from others? … you lack companionship? … left out?” (from 1 = *often* to 3 = *hardly ever or never*). The score of each item was reversed and the mean taken across items to reflect higher loneliness (range 1–3); Cronbach’s alpha was .73.

#### Dependent variables

*Cognitive performance.* Adopting the same approach as other HCAP-studies (Lee et al., 2025; Sutin et al., 2019; Weir et al., 2016), the cognitive tasks were grouped into five domains: (1) *Episodic memory* measured with the CERAD Word List Learning and Recall Task (immediate, delayed, recognition), the Wechsler Memory Scale - Logical Memory I, Long Story (immediate, delayed, recognition), and the Brave Man, Short Story (immediate, delayed). (2) *Speed-attention* measured with the Symbol Cancellation test, the Symbol-Digit Modalities test, Backward Counting, and Trail Making A and B. (3) *Visuospatial abilities* measured with Constructional Praxis (immediate and recall) and Raven’s matrices. (4) *Numeric reasoning* measured with the Health and Retirement Study (HRS) Number Series. (5) *Verbal fluency* measured with an animal fluency task. In addition, global cognitive function was measured with the Mini-Mental State Examination (MMSE; Folstein et al., 2001). For domains with multiple tasks, each task was standardized to a mean of 0 and a standard deviation of 1 and the mean taken across tasks into a composite score. Trails A and B were reversed (multiplied by -1) to match the direction of the other speed-attention measures. See supplementary material (Table S2).

*Informant-rated cognitive decline.* For each participant, one person who knew them well completed informant-rated measures of cognitive decline. The informant completed the Informant Questionnaire on Cognitive Decline in the Elderly (IQ-CODE; Jorm, 1994), the Community Screening Instrument for Dementia (CSI-D; Hall et al., 2000), the Blessed Dementia Rating Scale Part I (Morris et al., 1989), and the 10/66 (Prince et al., 2011). These scales were scored and *z*-standardized (*M* = 0, SD = 1), and the mean taken in the direction of greater informant-perceived cognitive decline. See supplementary material (Table S3).

#### Moderators

We tested cognitive status and depression as moderators of the associations. We used SHARE-HCAP classification of cognitive impairment (normal, mild and severe impairment) that was based on the approach described by Manly et al. (2022) and relied on diagnostic criteria from the National Institute on Aging and Alzheimer’s Association (Börsch-Supan et al., 2025). Using a deterministic algorithm, the SHARE-HCAP team classified participants as having a “severe impairment” if they scored 1.5 SD below the normative sample mean on at least two cognitive domains and their informant reported functional impairment. Participants with no impairment in any domain were classified as “normal”. Individuals were classified as having “mild cognitive impairment” if they had one impaired domain and functional impairment, or one impaired domain with either no informant report or self-reported memory concerns (Börsch-Supan et al., 2025).

Depression was assessed with the EURO-D scale (Gruber et al., 2024; Prince et al., 1999). Participants reported the presence or absence of 12 symptoms: depressed mood, pessimism, suicidality, guilt, troubles with sleep, loss of interest, irritability, change in appetite, fatigue, concentration, enjoyment, and tearfulness. Participants who endorsed 4 or more symptoms were classified as depressed (Gruber et al., 2024); this cutoff has been widely used in population-based studies to identify clinically relevant depressive symptomatology (Han et al., 2021). Additional exploratory analyses tested age, sex and education as moderators of the associations.

#### Covariates

Socio-demographic covariates were age in years, sex (0 = male, 1 = female), and education. SHARE used the 1997 International Standard Classification of Education (United Nations Educational, Scientific and Cultural Organization, 2003) to categorize and harmonize education statistics across European countries (from 0 = Pre-primary education to 6 = Secondary of tertiary education). Additional analysis controlled for health-related factors associated with risk of cognitive impairment and dementia (Livingston et al., 2024): activity limitations due to health problems (0 = no, 1 = yes), as evaluated by the Global Activity Limitations Index (van Oyen et al., 2006); diabetes (0 = no, 1 = yes); high blood pressure or hypertension (0 = no, 1 = yes); obesity (0 = no, 1 = yes, Body Mass Index ≥ 30); smoking status (0 = non-smoker, 1 = current/past smoker); physical inactivity (0 = vigorous or moderate activities, 1 = never vigorous nor moderate activities). For informant-rated cognitive decline, we controlled for characteristics of the informant and their relationship with the respondent: informant age, sex (0 = male, 1 = female), education (range 0–6), whether the informant was the spouse (0 = no, 1 = yes), and whether they lived with the respondent (0 = no, 1 = yes).

### Analytical strategy

We used linear mixed models to test the association between socio-emotional well-being and cognitive function using SPSS (version 29) because participants (Level 1) were nested within 5 countries (Level 2). We first fitted a null random-intercept-only model (without predictors) and calculated intraclass correlations (ICCs) for age, education, well-being and cognitive measures. ICCs indicated a small-to-moderate proportion of variance attributable to country-level clustering, supporting the use of multilevel modeling. We then fitted a series of models with each socio-emotional well-being variable entered separately as a predictor of the target outcome. Across models, continuous/ordinal variables were group-mean centered by subtracting their aggregate mean by country to remove country-level differences in the predictor and covariates. Model 1 examined each predictor (e.g., life satisfaction) of the outcome (e.g., MMSE) controlling for socio-demographic covariates. For informant-rated cognitive decline, Model 1 further controlled for characteristics of the informant. Model 2 added health-related factors associated with risk of dementia. Sensitivity analyses tested the robustness of the results: we retested Model 1 (a) controlling depression; (b) excluding participants who were classified with mild to severe cognitive impairment; and (c) entered all well-being variables together to evaluate their independent association with each cognitive measure. For scores based on performance on multiple cognitive tasks, supplemental analyses examined the association between well-being scales and each task to determine whether a specific task was driving domain-level associations. Moderation analyses tested whether the associations varied based on cognitive status, depression, age, sex, and education. For each moderator, we added the moderator variable (e.g., depression) and its interaction term with the well-being predictor (e.g., life satisfaction × depression) to the corresponding model, while controlling socio-demographic covariates and the main effect of the predictor (e.g., life satisfaction). For cognitive impairment, we dummy-coded cognitive status (reference = normal cognition) and entered interaction terms for both mild cognitive impairment and severe impairment within the same model. Because SPSS produces only unstandardized coefficients for linear mixed models, we *z*-standardized all continuous/ordinal variables and outcomes (*M* = 0, SD = 1) and re-ran the analysis to derive standardized coefficients (βs) of the associations (Snijders & Bosker, 2012). Significance was set to *p* < .01 (two-tail).

## Results

Descriptive statistics and zero-order correlations for the total sample and for each country are in Table 1 and Table S4.

### The association between socio-emotional well-being and cognitive measures

The linear mixed models indicated that all socio-emotional well-being scales were associated with global cognitive performance and informant-rated cognitive decline, after controlling for socio-demographic covariates and informant characteristics (Table 2): Participants who were more satisfied and found meaning in their lives and who were socially connected had better global cognitive performance (i.e., MMSE scores) and less informant-perceived decline, whereas those with higher loneliness had worse performance and greater informant-rated cognitive decline. The largest effects were observed for meaning in life (*β* = 0 .13 for global cognition; *β* = *−*.15 for informant-rated decline) and loneliness (*β* = *−*.11 for overall cognition; *β* = .21 for informant-rated decline). The effects were reduced in size but remained significant controlling for health-related covariates and depression; only the association between social connectedness and informant-rated cognitive decline was reduced to non-significance controlling for these covariates. When excluding participants with mild to severe cognitive impairment (total = 798), all dimensions, except social connectedness, remained associated at *p* < .01 with informant-rated cognitive decline, while only meaning in life and loneliness remained associated or showed trends toward significance (*p* < .05) with global cognitive performance. When entering all socio-emotional well-being factors as predictors of MMSE and informant scores, meaning in life and loneliness remained independently associated with both measures (see supplementary Table S5).

**Table 2.** Socio-emotional well-being associations with global cognition and informant-rated cognitive decline

	Global cognition					
	*N*	*B*	SE	*p*	Pseudo *R* ^2^	*β*
**Life satisfaction**						
Model 1	2,603	.222	.035	<.001	.257	.098
Model 2	2,559	.125	.035	<.001	.278	.055
Model 1 controlling for depression	2,444	.147	.035	<.001	.259	.065
Model 1 excluding impaired cases	1,664	.028	.028	.306	.242	.012
**Meaning in Life**						
Model 1	2,586	.706	.083	<.001	.269	.135
Model 2	2,541	.489	.082	<.001	.290	.093
Model 1 controlling for depression	2,424	.503	.081	<.001	.272	.096
Model 1 excluding impaired cases	1,653	.145	.064	.024	.248	.028
**Social Connectedness**						
Model 1	2,499	.342	.076	<.001	.270	.078
Model 2	2,450	.250	.072	<.001	.297	.057
Model 1 controlling for depression	2.334	.218	.071	.002	.271	.050
Model 1 excluding impaired cases	1,582	.087	.052	.092	.254	.020
**Loneliness**						
Model 1	2,634	−.888	.126	<.001	.279	−.115
Model 2	2,582	−.587	.122	<.001	.302	−.077
Model 1 controlling for depression	2,473	−.568	.130	<.001	.271	−.074
Model 1 excluding impaired cases	1,671	−.263	.093	.005	.249	−.034

	Informant-rated cognitive decline					
**Life satisfaction**						
Model 1	2,135	−.068	.011	<.001	.186	−.118
Model 2	2,097	−.030	.011	.005	.258	−.052
Model 1 controlling for depression	2,019	−.037	.011	<.001	.219	−.064
Model 1 excluding impaired cases	1,389	−.044	.009	<.001	.185	−.077
**Meaning in Life**						
Model 1	2,126	−.203	.025	<.001	.199	−.153
Model 2	2,087	−.131	.024	<.001	.270	−.099
Model 1 controlling for depression	2,008	−.136	.025	<.001	.234	−.103
Model 1 excluding impaired cases	1,380	−.074	.022	<.001	.181	−.056
**Social Connectedness**						
Model 1	2,036	−.077	.023	<.001	.194	−.069
Model 2	1,994	−.051	.021	.016	.272	−.046
Model 1 controlling for depression	1,915	−.028	.021	.188	.227	−.026
Model 1 excluding impaired cases	1,312	−.012	.017	.491	.173	−.011
**Loneliness**						
Model 1	2,172	.400	.039	<.001	.226	.206
Model 2	2,127	.299	.037	<.001	.294	.154
Model 1 controlling for depression	2,046	.281	.040	<.001	.247	.145
Model 1 excluding impaired cases	1,397	.309	.032	<.001	.229	.159

*Note:* Sample size (*N*) varies across models due to missing values. Model 1 controlled for age, sex, education, and informant-related characteristics for informant-rated cognitive decline; Model 2 further accounted for activity limitations, hypertension, diabetes, obesity, smoking and physical inactivity. Model 1 results remain consistent when residuals were weighted using SHARE-HCAP calibrated propensity score weights. Additional analyses controlled for depression and excluded individuals with mild to severe cognitive impairment (= impaired cases). All continues/ordinal predictive variables were centered by country prior to the analysis. Because SPSS produces only unstandardized coefficients for linear mixed models, we re-run the analysis using z-standardized values (*M* = 0, SD = 1) to derive standardized coefficients (βs) of the associations (Snijders & Bosker, 2012).

The socio-emotional well-being factors were also associated with specific cognitive domains (Table 3). Higher satisfaction and meaning in life were associated with better episodic memory, speed attention, visuo-spatial abilities, numeric reasoning and verbal fluency, controlling for age, sex, and education; these associations remained significant controlling for health-related covariates and depression, except for numeric reasoning. Higher social connectedness was associated with better performance across all domains except numeric reasoning; social connectedness remained associated with episodic memory, visuo-spatial ability, and verbal fluency but not with speed-attention controlling for health-related covariates and depression (Table 3). Higher loneliness was associated with worse performance in episodic memory, speed attention and verbal fluency, even when controlling health-related covariates and depression. For domains that were computed using multiple cognitive tasks, the domain-level associations with well-being dimensions were generally not driven by specific tasks (supplementary Table S6), except for social connectedness, where the association with speed-attention was primarily driven by the Symbol Cancellation and Symbol-Digit Modalities tasks and the association with visuospatial abilities was driven by the Constructional Praxis recall task (see supplementary Table S6). In sensitivity analyses that excluded individuals with mild to severe cognitive impairment, most associations were no longer significant; the exceptions were the associations between loneliness and episodic memory and speed attention, and life satisfaction and speed attention, which remained significant at *p* < .01. When entering all socio-emotional well-being factors as predictors of each cognitive domain (Table S5), meaning in life was independently associated with all cognitive domains except numeric reasoning. Of the other scales, life satisfaction was associated with speed-attention, loneliness was associated with episodic memory and verbal fluency, and social connectedness was associated with episodic memory, visuospatial abilities, and verbal fluency, accounting for the other well-being dimensions.

**Table 3.** Socio-emotional well-being associations with specific cognitive domains

	Episodic memory					
	*N*	*B*	SE	*p*	Pseudo *R* ^2^	*β*
**Life satisfaction**						
Model 1	2,599	.039	.008	<.001	.335	.080
Model 2	2,555	.023	.008	.005	.337	.046
Controlling for depression	2,442	.026	.008	.002	.332	.052
Excluding impaired cases	1,664	.014	.009	.120	.364	.028
**Meaning in life**						
Model 1	2,581	.112	.018	<.001	.341	.099
Model 2	2,536	.080	.019	<.001	.344	.070
Controlling for depression	2,422	.077	.019	<.001	.337	.067
Excluding impaired cases	1,653	.044	.021	.036	.366	.039
**Social connectedness**						
Model 1	2,494	.077	.016	<.001	.348	.081
Model 2	2,445	.067	.016	<.001	.353	.070
Controlling for depression	2,332	.060	.016	<.001	.339	.063
Excluding impaired cases	1,582	.017	.017	.318	.364	.017
**Loneliness**						
Model 1	2,631	−.172	.027	<.001	.350	−.103
Model 2	2,579	−.130	.027	<.001	.353	−.078
Controlling for depression	2,471	−.124	.029	<.001	.340	−.075
Excluding impaired cases	1,671	−.119	.030	<.001	.369	−.072

	Speed-attention					
**Life satisfaction**						
Model 1	2,580	.048	.008	<.001	.452	.090
Model 2	2,536	.024	.008	.002	.462	.045
Controlling for depression	2,433	.033	.008	<.001	.463	.062
Excluding impaired cases	1,659	.025	.008	.002	.520	.048
**Meaning in life**						
Model 1	2,562	.119	.018	<.001	.455	.098
Model 2	2,517	.074	.018	<.001	.468	.061
Controlling for depression	2,412	.082	.019	<.001	.466	.067
Excluding impaired cases	1,648	.035	.019	.068	.519	.029
**Social connectedness**						
Model 1	2,472	.043	.016	.006	.449	.042
Model 2	2,424	.030	.015	.050	.461	.030
Controlling for depression	2,321	.036	.016	.023	.463	.035
Excluding impaired cases	1,577	.013	.015	.410	.516	.012
**Loneliness**						
Model 1	2,608	−.137	.026	<.001	.463	−.077
Model 2	2,557	−.069	.026	.009	.476	−.039
Controlling for depression	2,458	−.071	.029	.014	.465	−.040
Excluding impaired cases	1,666	−.098	.028	<.001	.524	−.055

	Visuospatial abilities					
**Life satisfaction**						
Model 1	2,571	.043	.009	<.001	.354	.078
Model 2	2,528	.021	.009	.018	.363	.038
Controlling for depression	2,427	.027	.009	.003	.360	.048
Excluding impaired cases	1,658	.006	.008	.478	.418	.011
**Meaning in life**						
Model 1	2,553	.126	.021	<.001	.356	.098
Model 2	2,509	.080	.021	<.001	.365	.062
Controlling for depression	2,406	.082	.021	<.001	.359	.064
Excluding impaired cases	1,647	.008	.020	.693	.417	.006
**Social connectedness**						
Model 1	2,466	.072	.018	<.001	.359	.067
Model 2	2,418	.060	.018	<.001	.371	.055
Controlling for depression	2,316	.057	.016	.001	.362	.053
Excluding impaired cases	1,576	.034	.016	.029	.424	.032
**Loneliness**						
Model 1	2,603	−.131	.031	<.001	.360	−.070
Model 2	2,552	−.068	.031	.028	.371	−.036
Controlling for depression	2,453	−.069	.033	.034	.362	−.037
Excluding impaired cases	1,665	−.054	.028	.060	.420	−.028

	Numeric reasoning					
**Life satisfaction**						
Model 1	2,218	1.271	.401	.002	.238	.062
Model 2	2,189	.821	.412	.047	.240	.040
Controlling for depression	2,117	.950	.420	.024	.239	.046
Excluding impaired cases	1,539	.081	.460	.861	.260	.004
**Meaning in life**						
Model 1	2,198	2.690	.971	.006	.233	.057
Model 2	2,169	1.942	.989	.050	.237	.041
Controlling for depression	2,097	1.043	1.020	.307	.233	.022
Excluding impaired cases	1,527	−.068	1.107	.951	.259	−.001
**Social connectedness**						
Model 1	2,118	1.540	.791	.052	.241	.039
Model 2	2,086	1.388	.792	.080	.244	.035
Controlling for depression	2,017	1.394	.811	.086	.242	.035
Excluding impaired cases	1,461	−.712	.841	.397	.265	−.018
**Loneliness**						
Model 1	2,235	−3.009	1.439	.037	.240	−.044
Model 2	2,202	−1.821	1.466	.214	.241	−.026
Controlling for depression	2,134	−.728	1.570	.643	.238	−.011
Excluding impaired cases	1,545	−.283	1.582	.858	.263	−.004

	Verbal fluency					
**Life satisfaction**						
Model 1	2,596	.413	.078	<.001	.335	.084
Model 2	2,552	.264	.080	.001	.336	.054
Controlling for depression	2,443	.290	.082	<.001	.334	.059
Excluding impaired cases	1,664	.155	.100	.121	.370	.032
**Meaning in life**						
Model 1	2,579	1.104	.182	<.001	.342	.098
Model 2	2,534	.856	.186	<.001	.345	.076
Controlling for depression	2,423	.805	.192	<.001	.338	.071
Excluding impaired cases	1,653	.433	.236	.067	.372	.038
**Social connectedness**						
Model 1	2,492	.907	.158	<.001	.340	.096
Model 2	2,444	.836	.157	<.001	.344	.089
Controlling for depression	2,333	.715	.162	<.001	.333	.076
Excluding impaired cases	1,582	.263	.187	.159	.364	.028
**Loneliness**						
Model 1	2,628	−1.458	.266	<.001	.345	−.088
Model 2	2,577	−1.037	.271	<.001	.347	−.063
Controlling for depression	2,472	−1.029	.295	<.001	.338	−.062
Excluding impaired cases	1,671	−.709	.337	.036	.375	−.043

*Note:* Sample size (*N*) varies across models due to missing values. Model 1 controlled for age, sex, and education; Model 2 further accounted for activity limitations, hypertension, diabetes, obesity, smoking and physical inactivity. Model 1 results remain consistent when residuals were weighted using SHARE-HCAP calibrated propensity score weights. Additional analyses controlled for depression and excluded individuals with mild to severe cognitive impairment (= impaired cases). All continues/ordinal predictive variables were centered by country prior to the analysis. Because SPSS produces only unstandardized coefficients for linear mixed models, we re-run the analysis using *z*-standardized values (M = 0, SD = 1) to derive standardized coefficients (βs) of the associations (Snijders & Bosker, 2012).

### Moderation analysis

#### Cognitive status

In the current sample, 20.6% of participants (*n* = 546) were classified with mild cognitive impairment and 9.5% (*n* = 252) with severe impairment. Compared to participants without cognitive impairment, those with mild or severe impairment reported lower well-being and cognitive scores, along with greater variability (i.e., larger standard deviations) across most measures. Participants with severe impairment also showed lower well-being and cognitive function than those with mild impairment (see supplementary Note S1 and Table S7).

When formally testing cognitive status as a moderator of the associations, there was a significant interaction between severe cognitive impairment and meaning in life (*β* = .10, *p* = .009) and loneliness (*β* = *−*.11, *p* = .005) on MMSE performance (see supplementary Note 1 and Table S8); the interaction terms with life satisfaction and social connectedness showed only trends toward significance (*p* < .05). These results were consistent with the analysis that excluded individuals with cognitive impairment, which indicated that the associations with MMSE scores were primarily driven by the inclusion of individuals with severe cognitive impairment (supplementary Note 1 and Figure S1). As noted before, however, the associations for meaning in life and loneliness, although reduced in size, still remained significant or approached significance (*p* < .05) among participants without cognitive impairment.

In most cases, the remaining interactions between the well-being variables and cognitive status were non-significant when predicting informant-rated cognitive function and the specific cognitive domains (Note S1 and Table S8). There were a few notable exceptions: There was an interaction between loneliness and severe cognitive impairment predicting episodic memory (*β* = .10, *p* = .006) and speed attention (*β* = .12, *p* = .003). For these domains, loneliness was negatively associated with performance only among individuals without cognitive impairment (see Figure S1). There was also an interaction between cognitive status and life satisfaction predicting informant-rated cognitive decline and between cognitive status and social connectedness predicting speed-attention (Table S8); for life satisfaction, no association was observed with informant ratings for individuals with severe cognitive impairment, while, for social connectedness, a negative association emerged with speed attention for this impaired group (Figure S1).

#### Depression

About 25% (*n* = 655) respondents reported clinically relevant depressive symptomatology. Yet, few associations were moderated by depression. There was a significant interaction between depression and life satisfaction and depression and meaning in life on global cognition: the associations with MMSE scores were significant only among participants with depression (Note S1 and Figure S2). There was a significant interaction between depression and meaning in life on informant-rated cognitive decline that indicated a stronger association among participants with depression (Note S1 and Figure S2). No other interactions were significant.

#### Age, sex, and education

There were no interactions with sex or education. The few interactions with age were not consistent across either the well-being or cognitive measures. Results are detailed in supplementary material (Note S1 and Table S8).

## Discussion

This study investigated multiple dimensions of socio-emotional well-being in relation to cognitive performance and informant-rated cognitive decline with data from the HCAP sub-study of SHARE. Consistent with previous research (Lee et al., 2025; Sutin et al., 2024), socio-emotional well-being was associated with cognitive functioning in older adults. The strongest and most robust associations were observed for meaning in life (median *β* = .10) and loneliness (median *β* = −.09). These associations were generally attenuated, but remained statistically significant, adjusting for clinical (e.g., diabetes), behavioral (e.g., physical inactivity), and psychological (depression) risk factors for dementia. Moderation and sensitivity analyses suggested that some associations – particularly those with global cognition – may hinge on the inclusion of participants with cognitive impairment. Yet, some domain-specific associations (e.g., loneliness and episodic memory) were apparent only in individuals without impairment. Overall, evidence for moderation by cognitive status, depression and age was limited, and no moderation was observed for sex or education.

The current work supports evidence that loneliness is one critical dimension of socio-emotional well-being associated with poor cognitive performance (Lee et al., 2025). Across multiple models and sensitivity analyses, feeling lonely was consistently associated with lower scores on the MMSE, episodic memory, speed-attention, and verbal fluency tasks, as well as informant-rated cognitive decline. Models of loneliness and health (Cacioppo & Hawkley, 2009; Hawkley & Cacioppo, 2010) indicate that loneliness may affect cognitive function in multiple ways. For instance, individuals who feel lonely tend to withdraw from social and other cognitively stimulating activities, which increases their vulnerability to cognitive decline (Desai et al., 2025; Kim et al., 2020). They also tend to adopt unhealthy behaviors (e.g., be physically inactive) and experience depression, heightened stress/anxiety, and poor sleep (Christiansen et al., 2016; Dabiri et al., 2024), which may harm cognition over time. In line with prior work (Lee et al., 2025), the effect of loneliness on cognitive performance was attenuated but remained significant, accounting for depressive symptoms and health-related covariates, suggesting that loneliness may contribute to cognitive health through both direct and indirect pathways involving emotional and physical health.

An emerging finding in this study was that some associations between loneliness and cognitive outcomes varied by participants’ cognitive status. Moderation analyses indicated that while loneliness remained significantly associated with MMSE scores among individuals with normal cognition, the overall association with global cognitive performance was largely driven by participants with severe cognitive impairment, reinforcing prior evidence linking loneliness to risk of cognitive impairment and dementia (Livingston et al., 2024; Luchetti et al., 2024). A different pattern, however, emerged for domain-specific measures: for episodic memory and speed-attention, loneliness was associated with lower performance only among cognitively normal individuals. One possible interpretation is that, in cognitively healthy individuals, loneliness may act as a chronic stressor that disrupts processes such as attention regulation and memory encoding, whereas after cognitive impairment develops, loneliness may become more prevalent but less predictive of differences in memory and attention. A similar trend for episodic memory was observed in another HCAP study (Lee et al., 2025), though not for speed-attention.

In line with our hypothesis, individuals with higher scores on social connectedness – operationalized as having larger and more supportive social networks – had better cognitive performance across domains, particularly episodic memory and verbal fluency. Compared to loneliness, however, most associations were reduced to non-significance when controlling for health-related covariates and depression or excluding individuals with cognitively impairment, who are more likely to disengage from social activities (Zhu et al., 2023). This pattern suggests that subjective perceptions of isolation may be more relevant to cognitive function than objective measures of social isolation. Loneliness, for example, has an independent effect on cognition, even when controlling for the frequency of social contact and engagement in leisure or social activities (Luchetti et al., 2020; Sutin et al., 2023). The current study, however, did not examine specific characteristics of social relationships. Some evidence suggests that contact and support from friends, neighbors, or community members may be particularly important for maintaining cognitive function, potentially more so than support from family members (Meister & Zahodne, 2022). Notably, although social connectedness was lower among participants with cognitive impairment, most associations with this well-being dimension were not moderated by cognitive status.

As expected, life satisfaction and meaning in life were protective factors for cognitive functioning in older adults. Participants with greater satisfaction and meaning in life had better performance across cognitive measures and domains. These results align with a growing body of literature linking these well-being dimensions – particularly, a sense of purpose and goal orientation (Martela & Steger, 2016) – to reduced risk of cognitive impairment and better cognitive outcomes across diverse populations and cultural contexts (Sutin et al., 2024; Sutin et al., 2022; Sutin et al., 2024; Windsor et al., 2015; Zhu et al., 2022). Individuals with greater meaning or purpose in life may be more likely to engage in cognitively stimulating and health-promoting behaviors, thereby building cognitive reserve (Sutin et al., 2021). For example, in another HCAP study, having a sense of purpose in life was associated with informant-reported engagement in activities such as volunteering, which may help preserve cognitive function over time (Sutin et al., 2024). Similar to social connectedness, most associations with life satisfaction and meaning in life were non-significant excluding individuals with cognitive impairment. Formal tests of moderation by cognitive status revealed no significant interactions for most outcomes. There was an interaction between meaning in life and cognitive status on MMSE, suggesting that the association was primarily driven by individuals with severe cognitive impairment. Notably, associations with informant-rated cognitive decline remained significant among participants without impairment.

Taken together, the results underscore a key contribution of this study: although evidence for moderation was limited, some associations between well-being and cognitive measures varied by cognitive status. Approximately one-third of participants had mild to severe cognitive impairment, allowing meaningful group comparisons. Individuals with impairment reported lower well-being, poorer cognitive performance, and greater variability across measures, as reflected by larger standard deviations. It is important, however, to note that the SHARE classification of cognitive status relied on performance on HCAP tasks, including MMSE items (see Börsch-Supan et al., 2025). Because this classification is used solely to delineate cognitive groups, this approach is statistically acceptable for describing subgroup-specific associations. At the same time, the cognitive group definition partially incorporates the content of the HCAP battery, implying some degree of dependence between the moderator and the outcomes. Furthermore, the observed group differences should be interpreted taking into account the dynamic nature of both well-being and cognition, as research suggests both constructs may contribute to each other over time (e.g., Pfund et al., 2025). For instance, as cognitive abilities decline – whether due to age-related changes or emerging neuropathology – individuals may have fewer cognitive and emotional resources to maintain social connections, potentially leading to increased loneliness and reduced life satisfaction (e.g., Kwon & Yi, 2025). Due to the observational nature of the current study, it is not possible to determine directionality of the associations.

There was limited evidence for moderation by depression. For life satisfaction and meaning in life, the associations with global cognition and informant-rated cognitive decline were only significant, or stronger, among individuals with depression. The observed interactions suggest that these dimensions may serve as protective factors for those at elevated cognitive risk due to depression. However, no other significant interactions emerged between depression and well-being on cognitive performance. Moreover, depression may act more as a mediator (rather than a moderator) in the link between socio-emotional well-being and cognition (e.g., Dabiri et al., 2024).

Exploratory analyses indicated some moderation by age, but not by sex or education. For well-being dimensions, such as loneliness, some studies reported a stronger association with risk of cognitive impairment among relatively younger participants (60–79 years old), as compared to older groups (80 or older; Salinas et al., 2022), while others found loneliness associated with memory decline primarily in older participants (e.g., Yu et al., 2023). More recent meta-analytic evidence further suggests no consistent patterns of moderation by age, sex, or education (see Lee et al., 2025), consistent with the current findings. Taken together, the current results contribute to this literature by underscoring the generalizability of the association between socio-emotional well-being and cognition across multiple well-being dimensions, cognitive outcomes, and socio-demographic groups. Any observed moderation effects in this study should be interpreted with caution. As noted by Sherman and Pashler (2019), interaction effects are often difficult to replicate, and the likelihood of inconsistency increases with the number of interactions tested. Nonetheless, identifying whether certain socio-emotional factors are disproportionately associated with cognition in specific socio-demographic groups is an important direction for future research, especially given that most work has not formally examined age differences or other demographic factors that may shape well-being and increase cognitive risk.

An additional novel aspect of this study is the examination of the association between socio-emotional well-being and informant ratings of cognitive decline, which are considered necessary for a diagnosis of dementia (Chandrasekaran et al., 2026). Such ratings capture aspects of everyday cognitive ability that may not emerge in standardized testing and may reflect changes that individuals themselves do not recognize. Although some bias might be present, the current analysis accounted for informant characteristics and their relationship with participants. Individuals with higher socio-emotional well-being had better performance on objective cognitive tasks, and their informants similarly reported better cognitive functioning. These results offer converging evidence that socio-emotional well-being is linked with better cognitive abilities in daily life, as observed by close others.

The current study has several strengths, including the assessment of multiple dimensions of socio-emotional well-being, and use of a comprehensive cognitive battery to evaluate global and domain-specific cognitive function in a large, multi-national sample of older adults that ranged from healthy cognitive function to severe impairment. There are also limitations to be addressed in future studies. First, the study design with a single assessment of HCAP (a few months after the well-being measures) limits causal interpretations. Future work should replicate the observed associations using longitudinal data from upcoming HCAP waves. A longitudinal analysis is necessary for capturing within-person changes in well-being before and after the onset of cognitive impairment, as well as potential reciprocal associations between well-being and cognitive functioning. Second, this study used single items to assess life satisfaction and meaning in life. Although single items capture the constructs reasonably well (Cheung & Lucas, 2014; Sutin et al., 2022), multi-item scales are needed to examine specific facets of these dimensions (e.g., satisfaction across life domains). Third, methodological differences and unmeasured variables across SHARE countries may introduce variability or biases that were not accounted for in the current analysis. For example, data collection occurred between March and November 2022 – a period marked by varying COVID-19 mitigation measures across countries, which may have influenced participant responses and/or testing. Lastly, the sample was drawn exclusively from high-income European countries and future research should test whether the findings replicate in contexts with fewer economic resources to evaluate generalizability of the results across more diverse national and cultural contexts.

It is important to note that the observed associations are small when evaluated against traditional standards for effect size interpretation (e.g., Cohen’s guidelines). Nevertheless, given the complexity and multidetermined nature of cognitive health, even modest associations between socio-emotional well-being and cognition may hold substantive significance when considered alongside other contributing factors. Furthermore, small effects can yield meaningful consequences at the population level (Funder & Ozer, 2019), as such effects can accumulate over time and affect tens of millions of older adults within a nation or region.

In sum, this study highlights that having a sense of meaning and life satisfaction, less loneliness, and strong social connections may help support cognitive function. These findings align with the Positive Health framework that emphasizes the importance of individuals’ ability to adapt and self-manage in the face of emotional and social challenges to maintain overall functioning, including cognitive health. Interventions aimed at enhancing these well-being dimensions – whether individually or in combination – may be effective to support cognitive functioning across different stages of dementia. For instance, a recent scoping review by Joshi et al. (2024) found that technology-based social interventions reduce loneliness and improve social connections and quality of life among individuals with dementia and those at risk, even though these interventions did not yield measurable improvements in cognitive function. Additional work is needed to better understand how socio-emotional well-being contributes to cognitive health, identify which well-being dimensions and functional aspects are most effective intervention targets, and determine the optimal timing for such interventions across the dementia spectrum, including whether they are best implemented before or after the onset of cognitive impairment.

## Supporting information

Luchetti et al. supplementary material Luchetti et al. supplementary material

## Supplementary material

The supplementary material for this article can be found at https://doi.org/10.1017/S1355617726101933.
