# Supplementary material for: Socio-emotional well-being is associated with cognitive function and informant-rated cognitive decline: Results from the Harmonized Cognitive Assessment Protocol in Europe
Source: J Int Neuropsychol Soc. 2026 Mar 27:1–11. Online ahead of print. doi: 10.1017/S1355617726101933 (PMC13038402; doi:10.1017/S1355617726101933)
Supplement: Luchetti et al. supplementary material [file S1355617726101933sup001.docx]

Supplementary Material

**Socio-emotional well-being is associated with cognitive function and informant-rated cognitive decline: Results from the Harmonized Cognition Assessment Protocol in Europe**

**Table S1**

*Socio-emotional well-being measures and covariates*

| Variables | Description | Source |
| --- | --- | --- |
| Life satisfaction | Single question with responses from 0 to 10 (higher scores = higher satisfaction). | Wave 9 |
| Meaning in life | Single question from the Control-Autonomy-Pleasure-Self-realization (CAPS-12) scale. Responses 1 = Often, 2 =Sometimes, 3 = Rarely, 4 =Never. Scores were recorded in the direction of higher meaning. | Wave 9 |
| Social connectedness | The scale is operationalized and provided by SHARE (see Gruber et al., 2024). Scores (range 0-4) are in the direction of higher social connectedness. | Wave 9 |
| Loneliness | R-UCLA Loneliness scale (3-item). Responses 1= Often, 2=Some of the time, 3= Hardly ever or never. Scores were recorded in the direction of higher loneliness and the mean taken across items. | HCAP respondent file |
| Depression | EURO-D (12-item). The score is the sum of endorsed symptoms (yes/no) provided by SHARE. Cases with scores >= 4 (range 0-12) were classified as depressed. | HCAP respondent file |
| Cognitive Status | Classification of cognition status, pooled model - HRS-HCAP/Manly et al. 2022 criteria. Dummy coded: 1=mild impairment vs. 0=severe or no impairment; 1= severe impairment v. 0=mild or no impairment. | HCAP respondent file |
| Age | Age in years in 2022 (Wave 9). | Cover screen file |
| Sex | Biological sex at birth. Recoded as 1=female, 0=male. | HCAP respondent file |
| Education level | Respondent ISCED-97 level as provided by SHARE. Recoded 0 = None, 1 = ISCED-97 code 1, 2 = ISCED-97 code 2, 3 = ISCED-97 code 3, 4 = ISCED-97 code 4, 5= ISCED-97 code 5, 6 = ISCED-97 code 6, other values set as missing. | Wave 9 |
| Health-related limitations (Gali index) | Index score provided by SHARE (1=Limited, 0 = Not limited). | Wave 9 |
| Hypertension | High blood pressure or hypertension: ever diagnosed/currently having (1= yes, 0= no) | HCAP respondent file |
| Diabetes | Diabetes or high blood sugar: ever diagnosed/currently (1= yes, 0= no) | HCAP respondent file |
| Obesity | Body Mass Index (BMI) provided by SHARE. Recoded 1 = BMI>=30, 0 = BMI < 30. | Wave 9 |
| Ever smoked | Ever smoked daily (1= yes, 0= no). | Wave 9 |
| Physical inactivity | Score computed by SHARE (1= never vigorous nor moderate activities, 0=other). | Wave 9 |

**Table S2**

*Cognitive Measures*

| Domain | Measures | Test Description | Computing Score | Items |  |
| --- | --- | --- | --- | --- | --- |
| Global cognition | **Mini-Mental State Examination (MMSE)** | Max score 30. Orientation to time (5 points), orientation to place (5 points), 3-word immediate and delayed recall (6 points), counting or spelling backwards (5 points), object naming (2 point), repeat statement (2 points), 3-step task (3 points), follow command (1point), write sentence (1 point), draw picture (1 point). | We used the total score provided by the study. We selected participants with completion status = complete (1). | MMSE_score  MMSE_test |  |
| Episodic Memory | **CERAD Word List Memory** | Learning and immediate recall of 10 words (3 trials), score 0-30. | We used the total score provided by the study. We selected participants with completion status = complete (1) for any of the three trials. | WR103_test WR106_test WR109_test WR_immed_score |  |
|  |  |  |  |  |  |
|  |  | Delayed recall (1 trail), score 0-10. | We used the total score provided by the study. We selected participants with completion status = complete (1). | WR_delayed_score WR_delayed_test |  |
|  |  |  |  |  |  |
|  |  | Recognition (20 words), score 0-20. | We used the total score provided by the study. We selected participants with completion status = complete (1). | WRE_recognition_score WRE_recognition_test |  |
|  |  |  |  |  |  |
|  | **Logical Memory, Long Story** | Immediate Recall, score 0-25. Participants were given credit for exact answers (1 point each). | We used the total score provided by the study. We selected participants with completion status = complete (1). | LM_Story2_ImmedRecall_score LM_Story2_ImmedRecall_test |  |
|  |  | Immediate Recall, score 0-25. Participants were given credit for exact answers (1 point each). | We used the total score provided by the study. We selected participants with completion status = complete (1). | LM_Story2_DelayedRecall_score LM_Story2_DelayedRecall_test |  |
|  |  | Recognition, score 0-15. One point for each correct answer. | We used the total score provided by the study. We selected participants with completion status = complete (1). | LM_Recognition_score LM_Recognition_test |  |
|  | **Logical Memory, Short Story** | Immediate recall, score 0-6. Participants were given credit for exact answers (1 point each). | We used the total score provided by the study. We selected participants with completion status = complete (1). | LM_Story1_ImmedRecall_score LM_Story1_ImmedRecall_test |  |
|  |  | Delayed recall, score 0-6. Participants were given credit for exact answers (1 point each). | We used the total score provided by the study. We selected participants with completion status = complete (1). | LM_Story1_DelayedRecall_score LM_Story1_DelayedRecall_test |  |
| Speed-Attention | **Symbol Cancellation** | Point for each marked symbol. The score is derived from how many letters/symbols they mark given a time. | The study provides scores for three raters (correlation ~.99). We computed the mean across raters. We selected participants with completion status = complete (1). | hrsc001_correct_r1 hrsc001_correct_r2 hrsc001_correct_r3 SC_test |  |
|  | **Backward counting or naming** | Count backward from 100. The score is computed with the last number reached and the number of mistakes. | We used the total score provided by the study. We selected participants with completion status = complete (1). | BC_score BC_test |  |
|  | **Symbol Digit Modality Test** | Nine digits are presented associated with a symbol. The respondent has to fill in blank boxes with the symbol that correspond to each digit, according to the example given. The score is the highest number attempted minus the number of incorrect responses. | The study provides scores for three raters (correlation ~.99). We computed the mean across raters. We selected participants with completion status = complete (1). | SDMT_totcorr_r1 SDMT_totcorr_r2 SDMT_totcorr_r3 SDMT_test |  |
|  | **Trail Making A & B** | Trail A involves numbered circles, and the respondent is asked to draw a line linking the circles in numeric order (1, 2, 3, etc.). Trail B involves numbered circles and circles containing letters, and the respondent is asked to draw a line linking the numbers and letters alternately (1, A, 2, B, 3, C, etc.). The task is timed. | For Trail A and B, we used the total score provided by the study. We selected participants with completion status = complete (1). The score is reversed multiplying by -1. | TMTA_test  TMT_A_secs  TMTB_test TMT_B_secs |  |
| Visuospatial Ability | **Constructional Praxis (Immediate and Delayed Recall)** | Four sheets are typically presented: circle, rhombus, rectangle, and cube. The respondent must copy the corresponding figures. In the recall task, the respondent is asked to draw from memory the four figures. | For the immediate and delayed recall, we used the total score provided by the study. We select participants with completion status = complete (1) or cannot remember any of the 4 shapes (9) for delayed recall. | CP_score CP_test CP_delayed_score CP_delayed_test |  |
|  | **Raven's Matrixes** | Respondent is asked to identify the missing element that completes a pattern. The respondent is given 1 point for each pattern correctly identified. | We used the total score provided by the study. We selected participants with completion status (set A and B) = complete (1). | RV_total_score RV_setA_test RV_setB_test |  |
| Numeric Reasoning | **Number Series** | Respondent is presented with a series of numbers with one or two numbers missing. Participants are asked to identify the missing numbers. The test consists of a 6-item block adapted based on participants' performance. | We used the total score provided by the study. We selected participants with completion status (set 1 or set 2) = complete (1). | NS_Score12 NS_set1_test NS_set2_test |  |
| Language | **Verbal Fluency** | Animal naming. The score reflected the total number of correct animals named in 1 minute. | We used the total score provided by the study. We selected participants with completion status = complete (1). | RF_animalscore RF_animalscore_test |  |
|  |  |  |  |  |  |

**Table S3**

*Informant Measures*

| Measure | Test Description | Computing Score | Items |
| --- | --- | --- | --- |
| Jorm IQ-CODE | The 26-item version was administered. Items (e.g., how is the respondent at remembering things that have happened recently?) were on a 3-point scale: Improved, Not much changed, and Gotten worse. If changes occurred, the informant was asked for the degree of improvement/deterioration. For each item, a 5-point scale (5=much worse) was obtained. The total score was the mean across items (score range 1-5). | We used the total score provided by the study. | Jorm_score |
| CSI-D | Fifteen questions were asked to assess presence of certain symptoms or behaviors (e.g., difficulty in remembering things) with response options: Yes or No, or Yes, Yes, sometimes, No. The items were recoded as 1 (Yes, Yes, Sometimes) and 0 (No), and the sum was taken across items (range score 0-15). | We recoded the 15 items as 0/1 and computed the sum across the items. | hicd001_rs hicd006_rs hicd009_rs hicd010_rs hicd011_rs hicd012_rs hicd013_rs hicd014_rs hicd015_rs hicd016_rs hicd017_rs hicd018_rs hicd019_rs hicd020_rs hicd021_rs |
| Blessed Dementia Rating Scale – Part 1 | Eight questions (e.g., has there been a loss in remembering a short list of items, such as a shopping list?) were administered. For each question, responses were Severe loss, Some Loss, or No loss. The total score was the sum across items (range score 0-8). | We used the total score provided by the study. We selected participants with completion status = complete (1). | Blessed1_score Blessed1_test |
| 10/66 | Five questions (e.g., have difficulty performing household chores that [he / she] used to do?) with response options: Yes or No, or Yes, Yes, sometimes, No. The items were recoded as 1 (Yes, Yes, Sometimes) and 0 (No), and the sum taken across items (range score 0-5). | We recoded the 5 items as 0/1 and computed the sum across the items. | hitn003_rs hitn010_rs hitn001_rs hitn008_rs hitn007_rs |
| Informant Information | Age of the informant is reported in years. Sex is recoded as 1 = female and 0 = male. Education is reported in ISCED-97 levels. Relationship with the respondent is recoded as 1 = spouse and 0 = other. Living arrangements are recoded 1= living together with the respondent, 0 = not living together/other frequency of contact. | | |

**Table S4**

*Zero-order* *correlations*

| All sample (n = 2,650) | M | SD | 1 | 2 | 3 | 4 | 5 | 6 | 7 | 8 | 9 | 10 | 11 | 12 | 13 |
| --- | --- | --- | --- | --- | --- | --- | --- | --- | --- | --- | --- | --- | --- | --- | --- |
| 1. Age | 76.3 | 7.5 | 1 | -.267** | -.068** | -.174** | -.139** | .228** | -.376** | -.422** | -.496** | -.415** | -.252** | -.379** | .395** |
| 2. Education | 2.9 | 1.5 |  | 1 | .165** | .188** | .207** | -.181** | .387** | .423** | .482** | .464** | .399** | .427** | -.247** |
| 3. Life Satisfaction | 7.9 | 1.7 |  |  | 1 | .424** | .126** | -.370** | .193** | .183** | .207** | .157** | .141** | .189** | -.171** |
| 4. Meaning | 3.6 | 0.7 |  |  |  | 1 | .145** | -.343** | .266** | .243** | .261** | .220** | .148** | .243** | -.245** |
| 5. Social Connectedness | 2.1 | 0.9 |  |  |  |  | 1 | -.184** | .199** | .228** | .209** | .170** | .131** | .237** | -.166** |
| 6. Loneliness | 1.4 | 0.5 |  |  |  |  |  | 1 | -.263** | -.263** | -.277** | -.205** | -.156** | -.254** | .314** |
| 7. MMSE | 27.1 | 3.6 |  |  |  |  |  |  | 1 | .665** | .581** | .666** | .439** | .553** | -.635** |
| 8. Episodic Memory | 0.0 | 0.8 |  |  |  |  |  |  |  | 1 | .630** | .656** | .475** | .642** | -.562** |
| CERAD Imm. Word Recall | 16.0 | 5.9 | -.412** | .414** | .163** | .232** | .258** | -.240** | .607** | .816** | .632** | .582** | .421** | .633** | -.510** |
| CERAD Del. Word Recall | 4.7 | 2.8 | -.400** | .376** | .158** | .203** | .220** | -.235** | .539** | .803** | .585** | .539** | .396** | .593** | -.463** |
| CERAD Recognition | 17.5 | 3.1 | -.326** | .299** | .119** | .145** | .168** | -.216** | .547** | .672** | .470** | .483** | .292** | .428** | -.459** |
| Long Story Imm. Recall | 8.3 | 4.9 | -.313** | .321** | .126** | .186** | .142** | -.183** | .509** | .830** | .453** | .508** | .369** | .493** | -.440** |
| Long Story Del. Recall | 6.2 | 5.0 | -.330** | .330** | .134** | .196** | .144** | -.206** | .484** | .850** | .486** | .526** | .391** | .501** | -.409** |
| Long Story Recognition | 10.6 | 2.3 | -.199** | .184** | .134** | .117** | .096** | -.149** | .296** | .629** | .326** | .308** | .252** | .287** | -.239** |
| Short Story Imm. Recall | 3.7 | 1.6 | -.238** | .299** | .136** | .175** | .153** | -.174** | .520** | .760** | .422** | .485** | .328** | .456** | -.392** |
| Short Story Del. Recall | 2.6 | 1.9 | -.250** | .265** | .105** | .153** | .154** | -.160** | .451** | .767** | .405** | .474** | .319** | .415** | -.390** |
| 9. Speed Attention | -0.1 | 0.8 |  |  |  |  |  |  |  |  | 1 | .690** | .514** | .645** | -.486** |
| Symbol Cancellation | 33.9 | 14.9 | -.490** | .409** | .188** | .240** | .210** | -.238** | .502** | .558** | .827** | .597** | .391** | .590** | -.464** |
| Backward Counting | 28.2 | 11.3 | -.276** | .317** | .147** | .162** | .152** | -.203** | .374** | .398** | .723** | .406** | .355** | .452** | -.260** |
| Symbol Digit Modality Test | 30.1 | 14.2 | -.430** | .426** | .154** | .208** | .206** | -.222** | .475** | .546** | .813** | .564** | .448** | .547** | -.375** |
| Trail Making A | -71.9 | 50.1 | -.352** | .366** | .165** | .213** | .138** | -.169** | .506** | .491** | .786** | .554** | .363** | .479** | -.389** |
| Trail Making B | -145.7 | 70.4 | -.361** | .338** | .115** | .159** | .092** | -.173** | .395** | .430** | .798** | .473** | .380** | .465** | -.291** |
| 10. Visuospatial Ability | 0.0 | 0.9 |  |  |  |  |  |  |  |  |  | 1 | .545** | .568** | -.506** |
| Cons. praxis | 9.3 | 2.2 | -.278** | .354** | .124** | .180** | .118** | -.156** | .512** | .458** | .515** | .856** | .359** | .407** | -.348** |
| Cons. praxis, delayed recall | 7.0 | 3.4 | -.373** | .347** | .090** | .154** | .140** | -.142** | .522** | .593** | .561** | .853** | .414** | .488** | -.464** |
| Raven matrixes | 13.0 | 3.6 | -.378** | .475** | .182** | .200** | .159** | -.202** | .612** | .604** | .672** | .847** | .571** | .529** | -.437** |
| 11. Numeric Reasoning | 524.5 | 32.6 |  |  |  |  |  |  |  |  |  |  | 1 | .433** | -.278** |
| 12. Verbal Fluency | 20.2 | 7.8 |  |  |  |  |  |  |  |  |  |  |  | 1 | -.468** |
| 13. Informant Ratings | 0.0 | 0.9 |  |  |  |  |  |  |  |  |  |  |  |  | 1 |
| IQCODE | 3.2 | 0.4 | .371** | -.205** | -.120** | -.220** | -.146** | .252** | -.609** | -.522** | -.431** | -.469** | -.249** | -.424** | .913** |
| CSID | 2.7 | 2.9 | .321** | -.246** | -.164** | -.213** | -.165** | .303** | -.548** | -.516** | -.442** | -.446** | -.250** | -.446** | .884** |
| BlessedScale1 | 0.8 | 1.3 | .361** | -.225** | -.163** | -.238** | -.130** | .308** | -.619** | -.505** | -.435** | -.467** | -.238** | -.404** | .939** |
| 10 / 66 | 0.8 | 1.3 | .361** | -.202** | -.163** | -.211** | -.130** | .287** | -.530** | -.470** | -.417** | -.428** | -.222** | -.391** | .891** |

(continues)

| Czech Republic (n = 483) | M | SD | 1 | 2 | 3 | 4 | 5 | 6 | 7 | 8 | 9 | 10 | 11 | 12 | 13 |
| --- | --- | --- | --- | --- | --- | --- | --- | --- | --- | --- | --- | --- | --- | --- | --- |
| 1. Age | 76.4 | 6.7 | 1 | -.092* | .057 | -.123** | -.142** | .193** | -.317** | -.284** | -.428** | -.309** | -.203** | -.349** | .329** |
| 2. Education | 2.8 | 1.1 |  | 1 | .054 | .078 | .102* | -.004 | .217** | .207** | .280** | .349** | .309** | .280** | -.148** |
| 3. Life Satisfaction | 7.8 | 1.9 |  |  | 1 | .314** | .035 | -.398** | .124** | .132** | .116* | .145** | 0.043 | .184** | -0.097 |
| 4. Meaning | 3.6 | 0.7 |  |  |  | 1 | -.007 | -.262** | .197** | .133** | .232** | .166** | .121* | .213** | -.215** |
| 5. Social Connectedness | 1.9 | 0.8 |  |  |  |  | 1 | -.084 | .199** | .182** | .182** | .132** | .064 | .121** | -.177** |
| 6. Loneliness | 1.5 | 0.5 |  |  |  |  |  | 1 | -.190** | -.217** | -.259** | -.213** | -.141** | -.203** | .228** |
| 7. MMSE | 27.2 | 3.4 |  |  |  |  |  |  | 1 | .598** | .532** | .659** | .406** | .548** | -.652** |
| 8. Episodic Memory | 0.0 | 0.8 |  |  |  |  |  |  |  | 1 | .525** | .595** | .440** | .617** | -.583** |
| CERAD Imm. Word Recall | 16.0 | 5.6 | -.323** | .209** | .111* | .161** | .202** | -.221** | .541** | .755** | .521** | .496** | .394** | .605** | -.488** |
| CERAD Del. Word Recall | 4.7 | 2.7 | -.365** | .202** | .093* | .083 | .193** | -.191** | .516** | .764** | .517** | .509** | .394** | .587** | -.488** |
| CERAD Recognition | 17.4 | 2.9 | -.281** | 0.072 | .043 | .017 | .132** | -.179** | .467** | .648** | .422** | .424** | .267** | .439** | -.472** |
| Long Story Imm. Recall | 9.0 | 5.1 | -.209** | .204** | .101* | .134** | .125** | -.145** | .491** | .868** | .421** | .476** | .360** | .519** | -.504** |
| Long Story Del. Recall | 6.7 | 5.4 | -.226** | .204** | .094* | .109* | .105* | -.173** | .452** | .869** | .414** | .491** | .371** | .504** | -.461** |
| Long Story Recognition | 10.0 | 2.1 | -.102* | .223** | .003 | .071 | .091 | -.077 | .252** | .556** | .250** | .251** | .202** | .271** | -.270** |
| Short Story Imm. Recall | 3.8 | 1.8 | -.102* | .097* | .169** | .115* | .142** | -.169** | .436** | .777** | .328** | .457** | .313** | .413** | -.405** |
| Short Story Del. Recall | 2.8 | 2.2 | -.082 | .133** | .070 | .068 | .066 | -.114* | .398** | .792** | .289** | .457** | .289** | .354** | -.405** |
| 9. Speed Attention | -0.1 | 0.7 |  |  |  |  |  |  |  |  | 1 | .681** | .477** | .611** | -.537** |
| Symbol Cancellation | 33.7 | 13.5 | -.405** | .228** | .115* | .203** | .179** | -.208** | .494** | .440** | .804** | .561** | .341** | .565** | -.497** |
| Backward Counting | 24.1 | 9.7 | -.193** | .239** | .087 | .147** | .067 | -.131** | .186** | .274** | .660** | .377** | .310** | .370** | -.206** |
| Symbol Digit Modality Test | 29.1 | 11.1 | -.427** | .289** | .077 | .175** | .198** | -.196** | .483** | .495** | .818** | .537** | .417** | .562** | -.469** |
| Trail Making A | -65.6 | 36.9 | -.256** | .112* | .099* | .162** | .080 | -.133** | .459** | .396** | .770** | .524** | .322** | .433** | -.463** |
| Trail Making B | -140.7 | 63.4 | -.301** | .215** | .015 | .039 | .007 | -.137** | .301** | .309** | .798** | .418** | .357** | .350** | -.278** |
| 10. Visuospatial Ability | 0.2 | 0.8 |  |  |  |  |  |  |  |  |  | 1 | .570** | .561** | -.511** |
| Cons. praxis | 9.9 | 1.9 | -.113* | .262** | .133** | .083 | .098* | -.144** | .477** | .371** | .465** | .810** | .377** | .359** | -.326** |
| Cons. praxis, delayed recall | 8.0 | 3.3 | -.326** | .282** | .087 | .077 | .117* | -.178** | .559** | .572** | .585** | .854** | .423** | .536** | -.558** |
| Raven matrixes | 13.2 | 3.4 | -.285** | .319** | .123** | .181** | .134** | -.183** | .567** | .524** | .623** | .843** | .582** | .495** | -.370** |
| 11. Numeric Reasoning | 519.2 | 33.5 |  |  |  |  |  |  |  |  |  |  | 1 | .429** | -.289** |
| 12. Verbal Fluency | 20.8 | 7.6 |  |  |  |  |  |  |  |  |  |  |  | 1 | -.517** |
| 13. Informant Ratings | 0.0 | 1.0 |  |  |  |  |  |  |  |  |  |  |  |  | 1 |
| IQCODE | 3.2 | 0.4 | .354** | -.112* | -.067 | -.206** | -.157** | .189** | -.619** | -.540** | -.463** | -.454** | -.260** | -.453** | .910** |
| CSID | 2.6 | 2.9 | .238** | -.171** | -.085 | -.153** | -.154** | .192** | -.526** | -.527** | -.463** | -.446** | -.252** | -.461** | .874** |
| BlessedScale1 | 0.8 | 1.4 | .288** | -.111* | -.084 | -.203** | -.105 | .193** | -.698** | -.518** | -.476** | -.470** | -.266** | -.465** | .946** |
| 10 / 66 | 0.9 | 1.4 | .296** | -.118* | -.089 | -.186** | -.169** | .229** | -.570** | -.527** | -.530** | -.487** | -.262** | -.484** | .905** |

(continues)

| Denmark (n = 566) | M | SD | 1 | 2 | 3 | 4 | 5 | 6 | 7 | 8 | 9 | 10 | 11 | 12 | 13 |
| --- | --- | --- | --- | --- | --- | --- | --- | --- | --- | --- | --- | --- | --- | --- | --- |
| 1. Age | 75.2 | 7.5 | 1 | -.277** | -0.027 | -.163** | -.230** | .178** | -.335** | -.405** | -.559** | -.468** | -.301** | -.372** | .314** |
| 2. Education | 3.6 | 1.4 |  | 1 | .017 | .064 | .218** | -.077 | .213** | .258** | .349** | .354** | .319** | .293** | -.163** |
| 3. Life Satisfaction | 8.7 | 1.3 |  |  | 1 | .445** | .073 | -.283** | .051 | .060 | .157** | .081 | .088* | .057 | -.157** |
| 4. Meaning | 3.8 | 0.5 |  |  |  | 1 | .154** | -.317** | .112** | .143** | .194** | .127** | .099* | .089* | -.244** |
| 5. Social Connectedness | 2.3 | 0.9 |  |  |  |  | 1 | -.176** | .173** | .245** | .245** | .227** | .089* | .256** | -.157** |
| 6. Loneliness | 1.1 | 0.3 |  |  |  |  |  | 1 | -.128** | -.163** | -.270** | -.166** | -.093* | -.195** | .222** |
| 7. MMSE | 28.3 | 2.0 |  |  |  |  |  |  | 1 | .563** | .541** | .490** | .359** | .434** | -.478** |
| 8. Episodic Memory | 0.3 | 0.7 |  |  |  |  |  |  |  | 1 | .564** | .537** | .395** | .525** | -.517** |
| CERAD Imm. Word Recall | 18.3 | 5.6 | -.408** | .299** | .061 | .153** | .229** | -.153** | .522** | .788** | .582** | .505** | .374** | .542** | -.423** |
| CERAD Del. Word Recall | 5.9 | 2.7 | -.376** | .251** | .052 | .132** | .172** | -.125** | .456** | .761** | .514** | .452** | .321** | .466** | -.408** |
| CERAD Recognition | 18.5 | 2.4 | -.237** | .065 | -.023 | .038 | .117** | -.050 | .335** | .442** | .222** | .189** | .135** | .154** | -.310** |
| Long Story Imm. Recall | 9.8 | 4.8 | -.303** | .243** | .031 | .073 | .214** | -.122** | .438** | .824** | .436** | .422** | .339** | .448** | -.421** |
| Long Story Del. Recall | 8.0 | 5.0 | -.312** | .230** | .027 | .099* | .188** | -.137** | .440** | .847** | .441** | .443** | .351** | .422** | -.393** |
| Long Story Recognition | 11.5 | 2.2 | -.220** | .076 | .078 | .092* | .084 | -.087 | .267** | .667** | .295** | .312** | .254** | .199** | -.271** |
| Short Story Imm. Recall | 4.1 | 1.3 | -.187** | .156** | .038 | .109** | .148** | -.094* | .358** | .655** | .321** | .355** | .218** | .356** | -.266** |
| Short Story Del. Recall | 3.0 | 1.8 | -.219** | .133** | .066 | .130** | .227** | -.139** | .316** | .721** | .351** | .337** | .244** | .337** | -.365** |
| 9. Speed Attention | 0.4 | 0.7 |  |  |  |  |  |  |  |  | 1 | .622** | .464** | .619** | -.494** |
| Symbol Cancellation | 39.9 | 14.1 | -.523** | .327** | .188** | .196** | .264** | -.217** | .438** | .498** | .805** | .577** | .368** | .531** | -.446** |
| Backward Counting | 33.2 | 10.6 | -.247** | .175** | .072 | .100* | .110* | -.177** | .345** | .340** | .679** | .311** | .300** | .414** | -.271** |
| Symbol Digit Modality Test | 35.6 | 14.9 | -.441** | .311** | .116** | .103* | .206** | -.204** | .377** | .436** | .792** | .527** | .395** | .464** | -.288** |
| Trail Making A | -52.7 | 30.6 | -.443** | .227** | .100* | .122** | .212** | -.187** | .350** | .357** | .666** | .399** | .262** | .379** | -.363** |
| Trail Making B | -124.4 | 61.1 | -.410** | .232** | .128** | .176** | .134** | -.210** | .369** | .402** | .763** | .442** | .308** | .458** | -.340** |
| 10. Visuospatial Ability | 0.2 | 0.7 |  |  |  |  |  |  |  |  |  | 1 | .509** | .469** | -.409** |
| Cons. praxis | 9.7 | 1.9 | -.339** | .294** | .064 | .121** | .192** | -.131** | .408** | .364** | .462** | .836** | .362** | .336** | -.281** |
| Cons. praxis, delayed recall | 7.2 | 3.3 | -.404** | .252** | .085* | .099* | .165** | -.117** | .412** | .525** | .518** | .859** | .403** | .421** | -.422** |
| Raven matrixes | 14.3 | 2.6 | -.431** | .359** | .048 | .111** | .221** | -.127** | .385** | .436** | .577** | .792** | .487** | .383** | -.297** |
| 11. Numeric Reasoning | 535.1 | 27.7 |  |  |  |  |  |  |  |  |  |  | 1 | .347** | -.269** |
| 12. Verbal Fluency | 23.8 | 7.2 |  |  |  |  |  |  |  |  |  |  |  | 1 | -.412** |
| 13. Informant Ratings | -0.2 | 0.7 |  |  |  |  |  |  |  |  |  |  |  |  | 1 |
| IQCODE | 3.2 | 0.3 | .311** | -.152** | -.119* | -.179** | -.140** | .163** | -.491** | -.523** | -.453** | -.418** | -.240** | -.394** | .883** |
| CSID | 1.9 | 2.4 | .237** | -.119* | -.144** | -.256** | -.152** | .185** | -.393** | -.428** | -.420** | -.333** | -.238** | -.355** | .891** |
| BlessedScale1 | 0.4 | 0.8 | .254** | -.180** | -.172** | -.219** | -.073 | .214** | -.464** | -.419** | -.399** | -.293** | -.121* | -.288** | .908** |
| 10 / 66 | 0.7 | 1.1 | .274** | -.144** | -.164** | -.207** | -.137** | .228** | -.365** | -.416** | -.430** | -.325** | -.239** | -.351** | .869** |

(continues)

| France (n = 520) | M | SD | 1 | 2 | 3 | 4 | 5 | 6 | 7 | 8 | 9 | 10 | 11 | 12 | 13 |
| --- | --- | --- | --- | --- | --- | --- | --- | --- | --- | --- | --- | --- | --- | --- | --- |
| 1. Age | 77.2 | 8.2 | 1 | -.333** | -0.057 | -.225** | -.088* | .175** | -.365** | -.427** | -.559** | -.427** | -.321** | -.432** | .424** |
| 2. Education | 2.7 | 1.7 |  | 1 | .118** | .175** | .179** | -.072 | .370** | .432** | .493** | .542** | .439** | .375** | -.170** |
| 3. Life Satisfaction | 7.6 | 1.4 |  |  | 1 | .346** | .081 | -.306** | .084 | .089* | .130** | .060 | -.018 | .066 | .019 |
| 4. Meaning | 3.4 | 0.8 |  |  |  | 1 | .122** | -.364** | .252** | .231** | .253** | .252** | .124** | .244** | -.213** |
| 5. Social Connectedness | 2.1 | 0.8 |  |  |  |  | 1 | -.118** | .187** | .221** | .182** | .154** | .056 | .185** | -.150** |
| 6. Loneliness | 1.4 | 0.5 |  |  |  |  |  | 1 | -.117** | -.139** | -.146** | -.094* | -.076 | -.109* | .165** |
| 7. MMSE | 26.8 | 3.4 |  |  |  |  |  |  | 1 | .630** | .605** | .622** | .488** | .503** | -.585** |
| 8. Episodic Memory | -0.1 | 0.8 |  |  |  |  |  |  |  | 1 | .650** | .642** | .490** | .601** | -.546** |
| CERAD Imm. Word Recall | 16.2 | 5.6 | -.412** | .359** | .092* | .225** | .221** | -.129** | .570** | .828** | .638** | .558** | .431** | .571** | -.530** |
| CERAD Del. Word Recall | 4.6 | 2.8 | -.408** | .288** | .075 | .182** | .186** | -.141** | .502** | .797** | .542** | .494** | .398** | .515** | -.484** |
| CERAD Recognition | 18.2 | 2.6 | -.337** | .241** | .088* | .129** | .187** | -.083 | .518** | .657** | .435** | .422** | .321** | .367** | -.426** |
| Long Story Imm. Recall | 7.6 | 4.6 | -.277** | .396** | .094* | .208** | .150** | -.121** | .499** | .855** | .515** | .551** | .383** | .502** | -.376** |
| Long Story Del. Recall | 5.6 | 4.7 | -.324** | .377** | .098* | .188** | .153** | -.111* | .477** | .851** | .515** | .550** | .415** | .490** | -.364** |
| Long Story Recognition | 10.8 | 2.2 | -.226** | .264** | .099* | .130** | .084 | -.087 | .236** | .594** | .356** | .322** | .233** | .304** | -.134* |
| Short Story Imm. Recall | 3.6 | 1.5 | -.241** | .337** | .019 | .120** | .186** | -.096* | .471** | .763** | .453** | .459** | .362** | .407** | -.350** |
| Short Story Del. Recall | 2.5 | 1.9 | -.321** | .304** | .029 | .106* | .123** | -.108* | .440** | .775** | .460** | .492** | .350** | .412** | -.348** |
| 9. Speed Attention | 0.0 | 0.8 |  |  |  |  |  |  |  |  | 1 | .736** | .579** | .652** | -.489** |
| Symbol Cancellation | 34.4 | 15.2 | -.552** | .419** | .184** | .232** | .160** | -.178** | .459** | .567** | .845** | .595** | .470** | .569** | -.455** |
| Backward Counting | 29.0 | 10.4 | -.379** | .350** | .051 | .144** | .135** | -.071 | .467** | .457** | .770** | .549** | .407** | .511** | -.359** |
| Symbol Digit Modality Test | 30.8 | 13.6 | -.510** | .482** | .134** | .216** | .155** | -.168** | .537** | .618** | .857** | .644** | .506** | .555** | -.430** |
| Trail Making A | -70.6 | 45.2 | -.444** | .338** | .063 | .203** | .153** | -.112* | .540** | .503** | .800** | .616** | .467** | .493** | -.451** |
| Trail Making B | -145.7 | 71.6 | -.379** | .385** | .083 | .177** | .083 | -.091 | .439** | .461** | .840** | .533** | .451** | .510** | -.222** |
| 10. Visuospatial Ability | 0.0 | 0.8 |  |  |  |  |  |  |  |  |  | 1 | .608** | .587** | -.427** |
| Cons. praxis | 9.6 | 2.0 | -.285** | .424** | .089* | .240** | .118** | -.081 | .462** | .414** | .542** | .828** | .415** | .405** | -.290** |
| Cons. praxis, delayed recall | 7.3 | 3.5 | -.414** | .451** | .014 | .202** | .155** | -.099* | .580** | .647** | .655** | .868** | .506** | .539** | -.471** |
| Raven matrixes | 13.0 | 3.7 | -.371** | .511** | .050 | .178** | .097* | -.045 | .546** | .570** | .681** | .870** | .588** | .544** | -.334** |
| 11. Numeric Reasoning | 525.4 | 31.5 |  |  |  |  |  |  |  |  |  |  | 1 | .448** | -.268** |
| 12. Verbal Fluency | 20.4 | 7.3 |  |  |  |  |  |  |  |  |  |  |  | 1 | -.453** |
| 13. Informant Ratings | 0.0 | 0.8 |  |  |  |  |  |  |  |  |  |  |  |  | 1 |
| IQCODE | 3.2 | 0.4 | .407** | -.167** | .070 | -.206** | -.162** | .103* | -.588** | -.512** | -.459** | -.417** | -.316** | -.429** | .910** |
| CSID | 2.4 | 2.6 | .273** | -.144** | .013 | -.193** | -.130* | .147** | -.481** | -.489** | -.376** | -.330** | -.228** | -.384** | .837** |
| BlessedScale1 | 0.7 | 1.1 | .400** | -.116* | .028 | -.176** | -0.095 | .194** | -.492** | -.503** | -.438** | -.338** | -.202** | -.383** | .926** |
| 10 / 66 | 0.8 | 1.2 | .393** | -.135** | -.042 | -.183** | -.124* | .186** | -.471** | -.433** | -.420** | -.359** | -.165** | -.370** | .867** |

(continues)

| Germany (n = 546) | M | SD | 1 | 2 | 3 | 4 | 5 | 6 | 7 | 8 | 9 | 10 | 11 | 12 | 13 |
| --- | --- | --- | --- | --- | --- | --- | --- | --- | --- | --- | --- | --- | --- | --- | --- |
| 1. Age | 75.2 | 7.1 | 1 | -.079 | -0.054 | -.124** | -.114** | .155** | -.259** | -.381** | -.439** | -.346** | -.199** | -.315** | .305** |
| 2. Education | 3.7 | 1.1 |  | 1 | .129** | .087* | 0.073 | -0.073 | .230** | .274** | .229** | .365** | .378** | .187** | -.117* |
| 3. Life Satisfaction | 7.9 | 1.6 |  |  | 1 | .382** | .161** | -.362** | .169** | .146** | .192** | .160** | .175** | .164** | -.208** |
| 4. Meaning | 3.6 | 0.7 |  |  |  | 1 | .096* | -.284** | .150** | .191** | .192** | .146** | 0.065 | .155** | -.222** |
| 5. Social Connectedness | 2.4 | 0.8 |  |  |  |  | 1 | -.126** | .208** | .206** | .171** | .201** | .121** | .242** | -.167** |
| 6. Loneliness | 1.3 | 0.4 |  |  |  |  |  | 1 | -.101* | -.136** | -.071 | -.108* | -.081 | -.151** | .182** |
| 7. MMSE | 28.1 | 2.5 |  |  |  |  |  |  | 1 | .591** | .391** | .565** | .412** | .448** | -.553** |
| 8. Episodic Memory | 0.1 | 0.7 |  |  |  |  |  |  |  | 1 | .483** | .631** | .418** | .555** | -.455** |
| CERAD Imm. Word Recall | 17.8 | 5.2 | -.376** | .190** | .108* | .171** | .264** | -.080 | .518** | .772** | .514** | .516** | .317** | .507** | -.424** |
| CERAD Del. Word Recall | 5.3 | 2.6 | -.402** | .163** | .103* | .157** | .232** | -.135** | .454** | .770** | .474** | .472** | .281** | .492** | -.407** |
| CERAD Recognition | 18.3 | 2.4 | -.285** | .082 | .076 | .145** | .163** | -.122** | .462** | .610** | .348** | .401** | .222** | .325** | -.396** |
| Long Story Imm. Recall | 8.1 | 4.4 | -.278** | .301** | .110* | .150** | .105* | -.090* | .425** | .828** | .358** | .517** | .382** | .444** | -.331** |
| Long Story Del. Recall | 6.2 | 4.5 | -.269** | .270** | .094* | .166** | .101* | -.101* | .407** | .843** | .409** | .516** | .351** | .410** | -.294** |
| Long Story Recognition | 10.3 | 2.2 | -.190** | .111* | .113* | .093* | .031 | -.103* | .285** | .632** | .227** | .243** | .199** | .317** | -.197** |
| Short Story Imm. Recall | 3.8 | 1.4 | -.181** | .210** | .114** | .109* | .081 | -.079 | .411** | .691** | .253** | .445** | .304** | .342** | -.274** |
| Short Story Del. Recall | 2.8 | 1.8 | -.195** | .193** | .132** | .136** | .166** | -.087* | .430** | .738** | .359** | .506** | .313** | .350** | -.331** |
| 9. Speed Attention | 0.1 | 0.7 |  |  |  |  |  |  |  |  | 1 | .571** | .399** | .507** | -.452** |
| Symbol Cancellation | 36.0 | 13.1 | -.460** | .214** | .138** | .184** | .157** | -.031 | .349** | .437** | .800** | .499** | .263** | .466** | -.374** |
| Backward Counting | 29.9 | 9.6 | -.199** | .137** | .145** | .134** | .134** | -.079 | .275** | .288** | .695** | .303** | .253** | .397** | -.261** |
| Symbol Digit Modality Test | 32.3 | 12.4 | -.461** | .245** | .109* | .180** | .150** | -.062 | .379** | .487** | .774** | .502** | .401** | .446** | -.390** |
| Trail Making A | -63.2 | 40.3 | -.287** | .126** | .173** | .120** | .166** | -.057 | .377** | .368** | .723** | .350** | .233** | .344** | -.357** |
| Trail Making B | -144.1 | 67.0 | -.356** | .185** | .083 | .071 | .091 | -.044 | .315** | .363** | .793** | .413** | .307** | .409** | -.253** |
| 10. Visuospatial Ability | 0.0 | 0.7 |  |  |  |  |  |  |  |  |  | 1 | .494** | .471** | -.425** |
| Cons. praxis | 9.3 | 2.0 | -.190** | .294** | .069 | .111* | .139** | -.054 | .387** | .395** | .339** | .834** | .290** | .283** | -.246** |
| Cons. praxis, delayed recall | 6.9 | 3.2 | -.303** | .326** | .136** | .119** | .194** | -.069 | .418** | .600** | .484** | .857** | .386** | .406** | -.364** |
| Raven matrixes | 13.7 | 2.8 | -.340** | .317** | .165** | .057 | .151** | -.067 | .514** | .544** | .579** | .762** | .534** | .447** | -.383** |
| 11. Numeric Reasoning | 526.9 | 32.0 |  |  |  |  |  |  |  |  |  |  | 1 | .356** | -.276** |
| 12. Verbal Fluency | 22.0 | 7.2 |  |  |  |  |  |  |  |  |  |  |  | 1 | -.417** |
| 13. Informant Ratings | -0.1 | 0.8 |  |  |  |  |  |  |  |  |  |  |  |  | 1 |
| IQCODE | 3.2 | 0.3 | .282** | -.102* | -.170** | -.210** | -.166** | .126** | -.554** | -.429** | -.428** | -.421** | -.239** | -.355** | .903** |
| CSID | 2.4 | 2.4 | .256** | -0.069 | -.157** | -.174** | -.164** | .179** | -.443** | -.424** | -.377** | -.300** | -.230** | -.393** | .839** |
| BlessedScale1 | 0.6 | 1.0 | .273** | -0.099 | -.162** | -.172** | -.145** | .102* | -.521** | -.404** | -.372** | -.414** | -.251** | -.341** | .923** |
| 10 / 66 | 0.7 | 1.1 | .263** | -.142** | -.214** | -.195** | -.118* | .202** | -.436** | -.348** | -.369** | -.355** | -.234** | -.366** | .879** |

(continues)

| Italy (n = 535) | M | SD | 1 | 2 | 3 | 4 | 5 | 6 | 7 | 8 | 9 | 10 | 11 | 12 | 13 |
| --- | --- | --- | --- | --- | --- | --- | --- | --- | --- | --- | --- | --- | --- | --- | --- |
| 1. Age | 77.6 | 7.5 | 1 | -.296** | -.136** | -.118** | .006 | .316** | -.491** | -.540** | -.522** | -.506** | -.224** | -.393** | .499** |
| 2. Education | 1.7 | 1.2 |  | 1 | .143** | .093* | -.028 | -.137** | .330** | .357** | .421** | .421** | .267** | .317** | -.256** |
| 3. Life Satisfaction | 7.5 | 1.7 |  |  | 1 | .495** | .102* | -.285** | .225** | .160** | .121** | .173** | .226** | .134** | -.210** |
| 4. Meaning | 3.4 | 0.8 |  |  |  | 1 | .176** | -.303** | .276** | .206** | .145** | .209** | .147* | .181** | -.214** |
| 5. Social Connectedness | 1.8 | 0.8 |  |  |  |  | 1 | -.155** | .043 | .022 | -.050 | .018 | .088 | .037 | -.045 |
| 6. Loneliness | 1.6 | 0.6 |  |  |  |  |  | 1 | -.294** | -.256** | -.216** | -.215** | -.137* | -.232** | .394** |
| 7. MMSE | 24.8 | 5.0 |  |  |  |  |  |  | 1 | .726** | .551** | .734** | .435** | .593** | -.674** |
| 8. Episodic Memory | -0.6 | 0.8 |  |  |  |  |  |  |  | 1 | .604** | .698** | .493** | .612** | -.566** |
| CERAD Imm. Word Recall | 11.6 | 5.1 | -.500** | .331** | .155** | .171** | .057 | -.226** | .670** | .809** | .580** | .636** | .414** | .600** | -.554** |
| CERAD Del. Word Recall | 2.7 | 2.2 | -.402** | .296** | .143** | .140** | -.027 | -.189** | .557** | .794** | .531** | .587** | .392** | .523** | -.426** |
| CERAD Recognition | 15.1 | 3.9 | -.439** | .284** | .129** | .113* | -.032 | -.192** | .551** | .745** | .416** | .563** | .278** | .378** | -.463** |
| Long Story Imm. Recall | 7.0 | 5.1 | -.431** | .311** | .092* | .184** | .028 | -.241** | .611** | .833** | .462** | .531** | .384** | .497** | -.476** |
| Long Story Del. Recall | 4.3 | 4.7 | -.461** | .282** | .108* | .199** | .010 | -.258** | .538** | .834** | .480** | .541** | .418** | .504** | -.416** |
| Long Story Recognition | 10.3 | 2.6 | -.277** | .143* | .193** | .069 | .053 | -.141* | .422** | .692** | .329** | .410** | .276** | .295** | -.273** |
| Short Story Imm. Recall | 3.0 | 1.5 | -.378** | .279** | .104* | .210** | .010 | -.158** | .654** | .806** | .480** | .533** | .385** | .520** | -.436** |
| Short Story Del. Recall | 1.9 | 1.7 | -.363** | .224** | 0.057 | .168** | .041 | -.182** | .536** | .788** | .400** | .480** | .380** | .427** | -.389** |
| 9. Speed Attention | -0.7 | 0.9 |  |  |  |  |  |  |  |  | 1 | .692** | .432** | .520** | -.404** |
| Symbol Cancellation | 24.9 | 14.1 | -.488** | .326** | .078 | .147** | .020 | -.235** | .525** | .552** | .777** | .589** | .302** | .519** | -.452** |
| Backward Counting | 23.7 | 13.0 | -.288** | .243** | .091* | .070 | -.010 | -.153** | .362** | .356** | .697** | .405** | .314** | .360** | -.147** |
| Symbol Digit Modality Test | 21.2 | 14.3 | -.344** | .325** | .099* | .136** | .042 | -.192** | .424** | .457** | .723** | .499** | .339** | .414** | -.280** |
| Trail Making A | -113.0 | 69.8 | -.416** | .354** | .143** | .175** | -.112* | -.089 | .475** | .492** | .802** | .593** | .318** | .388** | -.320** |
| Trail Making B | -182.5 | 78.5 | -.371** | .334** | .101 | .138** | -.077 | -.200** | .369** | .386** | .749** | .410** | .310** | .323** | -.292** |
| 10. Visuospatial Ability | -0.6 | 1.1 |  |  |  |  |  |  |  |  |  | 1 | .549** | .539** | -.549** |
| Cons. praxis | 8.3 | 2.8 | -.400** | .331** | .152** | .193** | -.011 | -.196** | .600** | .545** | .581** | .888** | .381** | .465** | -.397** |
| Cons. praxis, delayed recall | 5.7 | 3.6 | -.426** | .355** | .095* | .185** | 0.048 | -.201** | .599** | .618** | .587** | .841** | .496** | .441** | -.465** |
| Raven matrixes | 10.6 | 4.3 | -.447** | .397** | .240** | .179** | -.040 | -.225** | .675** | .652** | .635** | .871** | .533** | .440** | -.532** |
| 11. Numeric Reasoning | 507.7 | 34.4 |  |  |  |  |  |  |  |  |  |  | 1 | .369** | -.432** |
| 12. Verbal Fluency | 14.0 | 5.8 |  |  |  |  |  |  |  |  |  |  |  | 1 | -.252** |
| 13. Informant Ratings | 0.3 | 1.1 |  |  |  |  |  |  |  |  |  |  |  |  | 1 |
| IQCODE | 3.3 | 0.5 | .434** | -.222** | -.168** | -.207** | -.041 | .358** | -.658** | -.538** | -.370** | -.506** | -.243** | -.439** | .929** |
| CSID | 3.9 | 3.7 | .450** | -.266** | -.203** | -.162** | -.058 | .377** | -.584** | -.502** | -.380** | -.502** | -.197** | -.426** | .908** |
| BlessedScale1 | 1.2 | 1.6 | .474** | -.190** | -.189** | -.225** | -.037 | .387** | -.622** | -.489** | -.343** | -.514** | -.195** | -.336** | .948** |
| 10 / 66 | 1.1 | 1.5 | .480** | -.239** | -.196** | -.204** | -.004 | .359** | -.610** | -.526** | -.362** | -.502** | -.219** | -.349** | .914** |

* *p* <. 0.05, ** *p* <.01 (two-tailed).

**Table S5**

*Sensitivity analysis*

|  | N | B | SE | p | Pseudo R^2^ | β |
| --- | --- | --- | --- | --- | --- | --- |
| ***Global Cognition*** |  |  |  |  |  |  |
| Life Satisfaction | 2,410 | .084 | .039 | .032 | .280 | .037 |
| Meaning in Life |  | .511 | .090 | <.001 |  | .098 |
| Social Connectedness |  | .171 | .070 | .015 |  | .039 |
| Loneliness |  | -.484 | .131 | <.001 |  | -.063 |
| ***Informant-rated Cognitive Decline*** |  |  |  |  |  |  |
| Life Satisfaction | 1,969 | -.023 | .012 | .061 | .216 | -.039 |
| Meaning in Life |  | -.128 | .028 | <.001 |  | -.096 |
| Social Connectedness |  | -.028 | .022 | .196 |  | -.026 |
| Loneliness |  | .301 | .042 | <.001 |  | .155 |
| ***Episodic Memory*** |  |  |  |  |  |  |
| Life Satisfaction | 2,408 | .018 | .009 | .044 | .350 | .036 |
| Meaning in Life |  | .073 | .021 | <.001 |  | .064 |
| Social Connectedness |  | .051 | .016 | .002 |  | .053 |
| Loneliness |  | -.108 | .030 | <.001 |  | -.065 |
| ***Speed-Attention*** |  |  |  |  |  |  |
| Life Satisfaction | 2,396 | .026 | .009 | .003 | .465 | .049 |
| Meaning in Life |  | .084 | .020 | <.001 |  | .069 |
| Social Connectedness |  | .026 | .016 | .096 |  | .026 |
| Loneliness |  | -.059 | .029 | .043 |  | -.033 |
| ***Visuospatial Abilities*** |  |  |  |  |  |  |
| Life Satisfaction | 2,389 | .023 | .010 | .021 | .360 | .041 |
| Meaning in Life |  | .094 | .023 | <.001 |  | .073 |
| Social Connectedness |  | .047 | .018 | .009 |  | .043 |
| Loneliness |  | -.052 | .034 | .123 |  | -.027 |
| ***Numeric Reasoning*** |  |  |  |  |  |  |
| Life Satisfaction | 2,067 | .959 | .455 | .035 | .241 | .047 |
| Meaning in Life |  | 1.388 | 1.084 | .201 |  | .029 |
| Social Connectedness |  | .915 | .806 | .256 |  | .023 |
| Loneliness |  | -1.122 | 1.593 | .481 |  | -.016 |
| ***Verbal Fluency*** |  |  |  |  |  |  |
| Life Satisfaction | 2,409 | .212 | .089 | .018 | .347 | .043 |
| Meaning in Life |  | .794 | .206 | <.001 |  | .070 |
| Social Connectedness |  | .640 | .161 | <.001 |  | .068 |
| Loneliness |  | -.790 | .299 | .008 |  | -.048 |

*Note.* Sample size (N) varies across models due to missing values*.* For each cognitive measure, we entered all socio-emotional well-being factors together as predictors of the outcome, controlling for socio-demographic covariates and informant-related characteristics (when relevant). All continues/ordinal variables were centered by the country prior to the analysis. Because SPSS produces only unstandardized coefficients for linear mixed models, we re-run the models using z-standardized values (M = 0, SD = 1) to derive standardized coefficients (βs) of the associations (Snijders & Bosker, 2012).

**Table S6**

*Socio-emotional well-being associations with individual cognitive tasks*

|  | ***Domain: Episodic Memory*** | | | | | |  |
| --- | --- | --- | --- | --- | --- | --- | --- |
|  | N | B | SE | p | Pseudo R^2^ | β |  |
| **Life Satisfaction 🡪** |  |  |  |  |  |  |  |
| CERAD Imm. Word Recall | 2,582 | .252 | .059 | <.001 | .349 | .068 |  |
| CERAD Del. Word Recall | 2,575 | .106 | .029 | <.001 | .320 | .060 |  |
| CERAD Recognition | 2,565 | .089 | .034 | .008 | .246 | .045 |  |
| Long Story Imm. Recall | 2,572 | .167 | .056 | .003 | .185 | .054 |  |
| Long Story Del. Recall | 2,557 | .171 | .057 | .003 | .204 | .054 |  |
| Long Story Recognition | 2,066 | .113 | .031 | <.001 | .121 | .078 |  |
| Short Story Imm. Recall | 2,586 | .071 | .018 | <.001 | .134 | .072 |  |
| Short Story Del. Recall | 2,557 | .062 | .023 | .007 | .121 | .051 |  |
| **Meaning in Life 🡪** | |  |  |  |  |  |  |
| CERAD Imm. Word Recall | | 2,562 | .784 | .137 | <.001 | .355 | .092 |
| CERAD Del. Word Recall | | 2,555 | .259 | .067 | <.001 | .322 | .064 |
| CERAD Recognition | | 2,543 | .172 | .078 | .028 | .246 | .038 |
| Long Story Imm. Recall | | 2,551 | .652 | .129 | <.001 | .191 | .092 |
| Long Story Del. Recall | | 2,536 | .653 | .131 | <.001 | .211 | .090 |
| Long Story Recognition | | 2,045 | .209 | .077 | .006 | .118 | .063 |
| Short Story Imm. Recall | | 2,566 | .199 | .042 | <.001 | .140 | .088 |
| Short Story Del. Recall | | 2,536 | .206 | .053 | <.001 | .125 | .074 |
| **Social Connectedness 🡪** |  |  |  |  |  |  |  |
| CERAD Imm. Word Recall | 2,477 | .727 | .118 | <.001 | .364 | .102 |  |
| CERAD Del. Word Recall | 2,471 | .244 | .058 | <.001 | .327 | .072 |  |
| CERAD Recognition | 2,461 | .157 | .067 | .020 | .262 | .042 |  |
| Long Story Imm. Recall | 2,467 | .334 | .110 | .002 | .194 | .056 |  |
| Long Story Del. Recall | 2,451 | .273 | .113 | .016 | .207 | .045 |  |
| Long Story Recognition | 1,963 | .076 | .062 | .220 | .119 | .027 |  |
| Short Story Imm. Recall | 2,480 | .110 | .036 | .002 | .148 | .058 |  |
| Short Story Del. Recall | 2,451 | .174 | .045 | <.001 | .131 | .075 |  |
| **Loneliness 🡪** | |  |  |  |  |  |  |
| CERAD Imm. Word Recall | | 2,611 | -1.012 | .200 | <.001 | .362 | -.081 |
| CERAD Del. Word Recall | | 2,608 | -.445 | .098 | <.001 | .328 | -.075 |
| CERAD Recognition | | 2,598 | -.482 | .115 | <.001 | .263 | -.073 |
| Long Story Imm. Recall | | 2,604 | -.942 | .188 | <.001 | .197 | -.090 |
| Long Story Del. Recall | | 2,590 | -1.033 | .191 | <.001 | .216 | -.097 |
| Long Story Recognition | | 2,070 | -.353 | .113 | .002 | .120 | -.072 |
| Short Story Imm. Recall | | 2,620 | -.267 | .061 | <.001 | .147 | -.081 |
| Short Story Del. Recall | | 2,590 | -.323 | .077 | <.001 | .132 | -.079 |
|  | | ***Domain: Speed-Attention*** | | | | | |
|  | | N | B | SE | p | Pseudo R^2^ | β |
| **Life Satisfaction 🡪** | |  |  |  |  |  |  |
| Symbol Cancellation | | 2,453 | .870 | .150 | <.001 | .383 | .094 |
| Backward Counting | | 2,546 | .438 | .126 | <.001 | .200 | .062 |
| Symbol Digit Modality Test | | 2,370 | .611 | .152 | <.001 | .361 | .069 |
| Trail Making A | | 2,438 | 2.615 | .543 | <.001 | .322 | .083 |
| Trail Making B | | 2,119 | 2.516 | .890 | .005 | .260 | .057 |
|  | | ***Domain: Speed-Attention*** | | | | | |
|  | | N | B | SE | p | Pseudo R^2^ | β |
| **Meaning in Life 🡪** | |  |  |  |  |  |  |
| Symbol Cancellation | | 2,436 | 2.141 | .349 | <.001 | .386 | .100 |
| Backward Counting | | 2,528 | 1.028 | .296 | <.001 | .200 | .063 |
| Symbol Digit Modality Test | | 2,352 | 1.622 | .356 | <.001 | .363 | .079 |
| Trail Making A | | 2,417 | 6.561 | 1.271 | <.001 | .326 | .091 |
| Trail Making B | | 2,096 | 6.481 | 2.109 | .002 | .259 | .064 |
| **Social Connectedness 🡪** | |  |  |  |  |  |  |
| Symbol Cancellation | | 2,348 | 1.242 | .301 | <.001 | .385 | .069 |
| Backward Counting | | 2,436 | .545 | .256 | .033 | .197 | .040 |
| Symbol Digit Modality Test | | 2,259 | 1.044 | .301 | <.001 | .363 | .061 |
| Trail Making A | | 2,325 | .492 | 1.087 | .651 | .319 | .008 |
| Trail Making B | | 2,013 | -.362 | 1.723 | .834 | .249 | -.004 |
| **Loneliness 🡪** | |  |  |  |  |  |  |
| Symbol Cancellation | | 2,478 | -2.370 | .523 | <.001 | .384 | -.075 |
| Backward Counting | | 2,570 | -1.352 | .434 | .002 | .209 | -.056 |
| Symbol Digit Modality Test | | 2,388 | -2.157 | .527 | <.001 | .364 | -.072 |
| Trail Making A | | 2,460 | -3.849 | 1.882 | .041 | .321 | -.036 |
| Trail Making B | | 2,131 | -10,833 | 3.118 | <.001 | .261 | -.073 |
|  | | ***Domain: Visuo-spatial Abilities*** | | | | | |
|  | | N | B | SE | p | Pseudo R^2^ | β |
| **Life Satisfaction 🡪** | |  |  |  |  |  |  |
| Cons. praxis | | 2,554 | .092 | .024 | <.001 | .212 | .066 |
| Cons. praxis, delayed recall | | 2,522 | .101 | .038 | .008 | .251 | .047 |
| Raven matrixes | | 2,508 | .192 | .037 | <.001 | .335 | .085 |
| **Meaning in Life 🡪** | |  |  |  |  |  |  |
| Cons. praxis | | 2,537 | .309 | .058 | <.001 | .216 | .097 |
| Cons. praxis, delayed recall | | 2,503 | .357 | .089 | <.001 | .255 | .072 |
| Raven matrixes | | 2,487 | .371 | .087 | <.001 | .330 | .071 |
| **Social Connectedness 🡪** | |  |  |  |  |  |  |
| Cons. praxis | | 2,446 | .147 | .050 | .003 | .207 | .055 |
| Cons. praxis, delayed recall | | 2,416 | .319 | .076 | <.001 | .261 | .077 |
| Raven matrixes | | 2,401 | .133 | .075 | .078 | .341` | .031 |
| **Loneliness 🡪** | |  |  |  |  |  |  |
| Cons. praxis | | 2,582 | -.293 | .086 | <.001 | .213 | -.063 |
| Cons. praxis, delayed recall | | 2,548 | -.387 | .131 | .003 | .256 | -.053 |
| Raven matrixes | | 2,538 | -.441 | .130 | <.001 | .339 | -.058 |

*Note.* For domain scores that included multiple cognitive tasks, we performed additional analysis to assess the association of socio-emotional well-being with each task. Each socio-emotional well-being scale was entered as predictor of the task, controlling for socio-demographic covariates. Sample size (N) varies across models due to missing values*.* All continues/ordinal variables were centered by the country prior to the analysis. Because SPSS produces only unstandardized coefficients for linear mixed models, we re-run the models using z-standardized values (M = 0, SD = 1) to derive standardized coefficients (βs) of the associations (Snijders & Bosker, 2012).

**Supplementary Note S1**

Based on the SHARE-HCAP classification of cognitive status, 798 participants had mild to severe cognitive impairment. Specifically, 546 were classified with mild cognitive impairment and 252 with severe impairment. Table S7 reports differences in well-being, cognitive measures, and covariates, across cognitive groups. Compared to unimpaired participants, those with mild to severe cognitive impairment were older, less educated, and more likely to report relevant depressive symptomatology, health-related limitations, physical inactivity, diabetes and obesity. Individuals with mild and severe cognitive impairment also reported lower scores on life satisfaction, meaning in life, social connectedness, and higher loneliness, compared to individuals with normal cognition (see Table S7 below). They also reported lower performance across cognitive measures, and higher informant-rated cognitive decline. Notably, individuals with impairment tended to have greater variability (i.e., larger standard deviations) in well-being and cognitive scores, compared to participants without cognitive impairment. Participants with severe cognitive impairment also reported lower well-being and worse cognitive function, compared to those with mild cognitive impairment (Table S7).

**Table S7**

*Differences by cognitive status*

| Variables | Normal  n = 1,674 | Mild imp.  n = 546 | Severe imp.  n = 252 | *F* or qui-squared stats. | Hedges' *g* |  |  |
| --- | --- | --- | --- | --- | --- | --- | --- |
|  |  |  |  |  | Normal vs. Mild imp. | Normal vs. Severe imp. | Mild vs. Severe imp. |
| Age (years) | 75.34 (7.23) | 76.88 (7.69) | 80.84 (6.91) | 64.71** | 0.21** | 0.77** | 0.53** |
| Sex (female) | 54.6 (914) | 50.0 (273) | 53.6 (135) | 3.50 | - | - | - |
| Education (range 0-6) | 3.08 (1.51) | 2.79 (1.45) | 2.33 (1.45) | 31.24** | 0.19** | 0.50** | 0.31** |
| Life satisfaction (range 0-10) | 8.11 (1.49) | 7.75 (1.64) | 7.43 (2.19) | 24.25* | 0.23** | 0.43** | 0.17* |
| Meaning in life (range 1-4) | 3.57 (0.62) | 3.52 (0.74) | 3.19 (0.91) | 53.78** | 0.23** | 0.72** | 0.41** |
| Social connectedness (range 0-4) | 2.22 (0.84) | 2.05 (0.85) | 1.77 (0.88) | 32.91** | 0.20** | 0.53** | 0.32** |
| Loneliness range (1-3) | 1.31 (0.45) | 1.41 (0.50) | 1.61 (0.63) | 47.36** | 0.22** | 0.65** | 0.37** |
| Depression (yes) | 20.7 (334) | 29.4 (147) | 58.4 (118) | 137.55** | - | - | - |
| Health-related limitations (yes) | 45.2 (756) | 62.5 (340) | 79.8 (201) | 132.79** | - | - | - |
| Hypertension (yes) | 50.9 (851) | 54.3 (296) | 50.4 (125) | 2.11 | - | - | - |
| Diabetes (yes) | 15.3 (256) | 23.3 (127) | 22.6 (56) | 22.29** | - | - | - |
| Obesity (yes) | 21.9 (361) | 28.9 (154) | 23.7 (57) | 11.09** | - | - | - |
| Ever smoked (yes) | 43.5 (728) | 40.7 (222) | 35.9 (90) | 5.85 | - | - | - |
| Physical inactivity (yes) | 11.5 (192) | 20.9 (114) | 49.4 (124) | 224.21** | - | - | - |
| MMSE (range 0-30) | 28.40 (1.83) | 25.99 (3.58) | 21.31 (5.43) | 738.39** | 1.01** | 2.73** | 1.10** |
| Episodic memory (composite) | 0.27 (0.64) | -0.39 (0.67) | -1.09 (0.59) | 611.77** | 1.02** | 2.14** | 1.08** |
| Speed-attention (composite) | 0.22 (0.68) | -0.40 (0.79) | -1.08 (0.88) | 409.87** | 0.87** | 1.84** | 0.83** |
| Visuo-spatial abilities (composite) | 0.32 (0.63) | -0.44 (0.86) | -1.26 (1.00) | 607.07** | 1.09** | 2.30** | 0.91** |
| Numeric reasoning (HRS series) | 531.74 (28.75) | 512.27 (33.14) | 496.14 (36.79) | 133.81** | 0.65** | 1.21** | 0.47** |
| Verbal fluency (animal naming) | 22.80 (7.19) | 17.45 (6.48) | 11.03 (5.29) | 383.17** | 0.76** | 1.69** | 1.05** |
| Informant-rated cognitive  decline (composite) | -0.29 (0.53) | 0.06 (0.79) | 1.66 (1.10) | 862.99** | 0.57** | 3.00** | 1.78* |

*Note.* Means (and standard deviations) are reported for continues/ordinal scales; percentages (n) are reported for categorical/binary variables. 178 participants were missing cognitive status. *F* or qui-squared tests are reported; Hedges' *g*s are computed to compare continues/ordinal scale differences between groups. Hedges' *g* of 0.2 = small; 0.5 = medium; 0.8 = large differences. **p*<. 0.05, ***p* <.01.

**Moderation by Cognitive Status**

As shown in Table S8, when formally testing cognitive status (normal [ref. category], mild or severe cognitive impairment) as a moderator of the association between socio-emotional well-being and MMSE, the interaction terms involving severe cognitive impairment were significant for meaning in life and loneliness (p < .01) and approached significance for life satisfaction and social connectedness (p < .05). As illustrated in Figure S1 (Panel (a)), and in line with the results from the analysis that excluded individuals with cognitive impairment, the observed associations with MMSE scores were primarily driven by the inclusion of individuals with severe cognitive impairment. In stratified-group analysis, the associations of meaning in life and loneliness with MMSE were attenuated among individuals with normal cognition and appeared stronger among participants with severe impairment, although subgroup associations did not reach statistical significance—likely due to the smaller size of the groups with mild (n = 546) or severe cognitive impairment (n = 252). Specifically, Meaning in Life → Global Cognition BY Cognitive Status: β _no impairment_ = .03 (p =.024), β _mild impairment_ = .04 (p =.179), β _severe impairment_ = .11 (p =.063); Loneliness → Global Cognition BY Cognitive Status: β _no impairment_ = -.03 (p =.005), β _mild impairment_ = -.01 (p =.724), β _severe impairment_ = -.08 (p =.228).

The remaining interactions between the well-being variables and cognitive status were non-significant when predicting informant-rated cognitive function and specific cognitive domains, with a few notable exceptions. Loneliness interacted with cognitive status when predicting episodic memory and speed attention (see Table S8). As illustrated in Figure S1 (Panel (c) and (d)), and indicated by stratified analysis, a negative association emerged between loneliness and episodic memory and speed-attention for participants without cognitive impairment, but not for individuals with mild to severe cognitive impairment. Specifically. Loneliness → Episodic Memory BY Cognitive Status: β _no impairment_ = -.07 (p <.001), β _mild impairment_ = .02 (p =.396), β _severe impairment_ = .02 (p =.506); Loneliness → Speed-attention BY Cognitive Status: β _no impairment_ = -.05 (p <.001), β _mild impairment_ = .02 (p =.576), β _severe impairment_ = .04 (p =.329).

Life satisfaction interacted with cognitive status when predicting informant-rated cognitive decline (Table S8). As shown in Figure S1 (Panel (b)), and in group-stratified analyses, life satisfaction was negatively associated with informant-rated decline in participants with normal or mild cognitive impairment, but not those with severe impairment, which had higher informant-rated decline. Specifically, Life Satisfaction → Informant-rated Cognitive Decline BY Cognitive Status: β _no impairment_ = -.08 (p <.001), β _mild impairment_ = -.08 (p =.018), β _severe impairment_ = .01 (p =.868).

Social connectedness also interacted with cognitive status when predicting speed-attention (Table S8). As shown in Figure S1 (Panel (d)), and in group-stratified analyses, there was a negative association among individuals with severe cognitive impairment. Specifically, Social Connectedness → Speed-attention BY Cognitive Status: β _no impairment_ = .01 (p =.410), β _mild impairment_ = .04 (p =.258), β _severe impairment_ = -.13 (p =.029). This group performed worse in speed-attention at higher level of social connectedness, possibly because individuals with severe impairment needed assistance and had a larger social network, due to their needs.

**Moderation by Depression**

As shown in Table S8, only a few associations were moderated by presence/absence of depressive symptomatology. When predicting global cognition, there was a significant interaction between depression and life satisfaction and depression and meaning in life. As illustrated in Figure S2, and indicated by stratified-group analyses, the association between these well-being dimensions and the MMSE was significant only among individuals with depression. Specifically, Life Satisfaction → Global Cognition BY Depression: β _depressed_ = .14 (p <.001), β _no-depressed_ = .01 (p =.724); Meaning in Life → Global Cognition BY Depression: β _depressed_ = .17 (p <.001), β _no-depressed_ = .03 (p =.083). When predicting informant-rated cognitive decline, there was a significant interaction between depression and meaning in life, which indicated a stronger association among individuals with depression (β _depressed_ = -.15, p <.001; β _no-depressed_ = -.06, p =.011). These results suggest that having meaning in life or higher life satisfaction could be protective in individuals with depression, who are known to be at risk for poor cognitive outcomes. Yet, no other significant interactions were observed between depression and well-being dimensions when predicting performance on cognitive domains.

**Moderation by Age, Sex, and Education**

As shown in Table S8, age moderated only a few associations, while sex and education did not moderate any. No consistent pattern of age moderation emerged across the cognitive and well-being measures.

When predicting global cognition, there was a significant interaction between age and meaning in life and age and social connectedness. Stratified-group analyses indicated that the association between these well-being dimensions and the MMSE was stronger or significant only among participants who were older than the mean-level age of their country. Specifically, Meaning in Life → Global Cognition BY Age: β _older_ = .16 (p <.001), β _younger_ = .10 (p <.001); Social Connectedness → Global Cognition BY Age: β _older_ = .16 (p <.001), β _younger_ = .03 (p =.047). When predicting informant-rated cognitive decline, there was a significant interaction between age and social connectedness, but not other well-being dimensions; the association between social connectedness and informant ratings was significant only among older participants (β _older_ = -.15, p <.001; β _younger_ = -.02, p =.320).

For the cognitive domains, there was a significant interaction between age and loneliness when predicting speed-attention and visuospatial ability, and age and life satisfaction when predicting numeric reasoning and verbal fluency. As indicated in stratified-group analyses, the association between these well-being variables and the cognitive domains was apparent only among individuals who were relatively younger than their country-mean age. Specifically, Loneliness → Speed-attention BY Age: β _older_ = -.03 (p =.192), β _younger_ = -.13 (p <.001); Loneliness → Visuo-spatial Ability BY Age: β _older_ = -.03 (p =.293), β _younger_ = -.11 (p <.001); Life Satisfaction → Numeric Reasoning BY Age: β _older_ = -.03 (p =.398), β _younger_ = .13 (p <.001); Life Satisfaction → Verbal Fluency BY Age: β _older_ = .05 (p =.017), β _younger_ = .11 (p <.001).

**Table S8**

*Moderation*

| **Moderator:** | Cognitive Status [ref. normal cognition] | | | | Depression | | Age | | Sex | | Education | |
| --- | --- | --- | --- | --- | --- | --- | --- | --- | --- | --- | --- | --- |
|  | Mild Impairment | | Severe Impairment | |  |  |  |  |  |  |  |  |
| ***Global Cognition*** | β | p | β | p | β | p | β | p | β | p | β | p |
| Life Satisfaction | .043 | .187 | .086 | .018 | **.137** | **<.001** | .020 | .201 | -.003 | .929 | -.005 | .764 |
| Meaning in Life | .052 | .112 | **.099** | **.009** | **.151** | **<.001** | **.046** | **.002** | .058 | .065 | -.010 | .504 |
| Social Connectedness | .020 | .566 | .094 | .040 | .055 | .125 | **.046** | **.008** | .022 | .530 | -.027 | .115 |
| Loneliness | -.010 | .768 | **-.107** | **.005** | -.067 | .048 | .019 | .212 | .058 | .078 | -.027 | .090 |
| ***Informant ratings*** | β | p | β | p | β | p | β | p | β | p | β | p |
| Life Satisfaction | .004 | .916 | **.106** | **.007** | -.078 | .039 | .038 | .044 | .077 | .045 | -.044 | .024 |
| Meaning in Life | -.036 | .315 | .011 | .785 | **-.104** | **.006** | -.019 | .269 | -.024 | .532 | -.032 | .078 |
| Social Connectedness | .015 | .682 | -.068 | .153 | -.065 | .136 | **-.059** | **.004** | -.051 | .207 | .023 | .284 |
| Loneliness | -.026 | .457 | -.071 | .080 | .094 | .020 | -.001 | .956 | -.040 | .309 | .010 | .590 |
| ***Episodic Memory*** | β | p | β | p | β | p | β | p | β | p | β | p |
| Life Satisfaction | .007 | .845 | -.086 | .023 | .023 | .494 | -.021 | .193 | -.010 | .754 | .017 | .308 |
| Meaning in Life | .003 | .939 | -.096 | .013 | .022 | .518 | .010 | .500 | -.012 | .708 | .003 | .840 |
| Social Connectedness | -.030 | .371 | .060 | .183 | .014 | .719 | .013 | .439 | .045 | .175 | .002 | .913 |
| Loneliness | **.090** | **.007** | **.104** | **.006** | .001 | .981 | .039 | .010 | .045 | .161 | -.022 | .158 |
| ***Speed-Attention*** | β | p | β | p | β | p | β | p | β | p | β | p |
| Life Satisfaction | .001 | .732 | -.060 | .096 | -.023 | .453 | -.035 | .016 | -.064 | .029 | -.013 | .373 |
| Meaning in Life | .043 | .188 | -.020 | .601 | -.003 | .923 | -.009 | .514 | -.039 | .180 | -.004 | .781 |
| Social Connectedness | .016 | .636 | **-.125** | **.005** | .020 | .558 | -.010 | .541 | .015 | .632 | -.003 | .852 |
| Loneliness | .068 | .035 | **.117** | **.003** | .023 | .467 | **.051** | **<.001** | .029 | .324 | -.014 | .310 |
| ***Visuospatial Abilities*** | β | p | β | p | β | p | β | p | β | p | β | p |
| Life Satisfaction | .020 | .536 | .030 | .420 | .066 | .044 | -.036 | .025 | -.005 | .866 | .008 | .620 |
| Meaning in Life | .043 | .187 | .049 | .210 | .036 | .272 | .023 | .127 | .038 | .237 | -.007 | .647 |
| Social Connectedness | -.027 | .414 | .011 | .802 | .022 | .545 | .023 | .177 | .036 | .279 | -.012 | .483 |
| Loneliness | .066 | .041 | -.006 | .883 | -.029 | .395 | **.049** | **.002** | .017 | .614 | -.037 | .018 |
| ***Numeric Reasoning*** | β | p | β | p | β | p | β | p | β | p | β | p |
| Life Satisfaction | .040 | .383 | .113 | .064 | -.035 | .402 | **-.062** | **.002** | -.075 | .057 | .005 | .802 |
| Meaning in Life | .043 | .391 | .119 | .057 | -.032 | .467 | .008 | .670 | -.027 | .507 | -.002 | .926 |
| Social Connectedness | .085 | .071 | .050 | .501 | .120 | .012 | .030 | .142 | .043 | .284 | -.024 | .223 |
| Loneliness | .001 | .982 | -.099 | .184 | .058 | .196 | .042 | .036 | .059 | .157 | .006 | .737 |
| **Moderator:** | Cognitive Status [ref. normal] | | | | Depression | | Age | | Sex | | Education | |
|  | Mild Impairment | | Severe Impairment | |  |  |  |  |  |  |  |  |
| ***Verbal Fluency*** |  |  |  |  |  |  | β | p | β | p | β | p |
| Life Satisfaction | .012 | .751 | -.036 | .376 | .030 | .375 | **-.043** | **.007** | .021 | .520 | .029 | .079 |
| Meaning in Life | -.006 | .865 | -.052 | .221 | .020 | .551 | .004 | .784 | .065 | .041 | .013 | .414 |
| Social Connectedness | .050 | .187 | .032 | .513 | .050 | .190 | .017 | .323 | .007 | .827 | .002 | .885 |
| Loneliness | .026 | .475 | .007 | .869 | -.042 | .236 | .020 | .191 | .014 | .654 | -.008 | .595 |

*Note.* Each cognitive measure was regressed on socio-emotional well-being factors, the moderator of interest (e.g., depression), and their interaction term (life satisfaction x depression), controlling for age, sex, and education. For cognitive impairment, we entered a dummy coded cognitive status variable (ref. normal cognition) and entered interaction terms with mild cognitive impairment and severe impairment in the same model. For a better visualization of the table, we report only z-standardized coefficients (βs) and p-values of the interactions.

**Figure S1**

*Moderation by Cognitive Status*

****Panel (a)**

DV = Global cognitive function

**Panel (b)**

DV = Informant-rated Cognitive Decline

**Panel (c)**

DV = Episodic Memory

**Panel (d)**

DV = Speed-attention

*Note.* Significant interactions with cognitive status were plotted in Stata 19 using estimated marginal means (*margins* and *marginsplot*). Please note that all statistical analyses in the manuscript were performed in SPSS.

**Figure S2**

*Moderation by Depression*

**Panel (a)**

DV = Global cognitive function

**

**Panel (b)**

VD= Informant-rated Cognitive Decline

**

*Note.* Significant interactions with depression were plotted in Stata 19 using estimated marginal means (*margins* and *marginsplot*). Please note that all statistical analyses in the manuscript were performed in SPSS.
